# Development and initial validation of a computerized adaptive test prototype for ECG interpretation using item response theory

**DOI:** 10.1371/journal.pone.0344341

**Published:** 2026-07-17

**Authors:** Shinji Inaba, Kazumichi Yamamoto, Tomohiro Kaga, Hiroshi Kawakami, Mohammad Zahidul Islam, Muhammad Wannous, Masatsugu Sakata, Toshi A. Furukawa, Osamu Yamaguchi

**Affiliations:** 1 Department of Cardiology, Pulmonology, Hypertension and Nephrology, Ehime University Graduate School of Medicine, Toon, Japan; 2 Department of Health Promotion and Human Behavior, Kyoto University Graduate School of Medicine/School of Public Health, Kyoto, Japan; 3 Institute for Airway Disease, Hyogo, Japan; 4 Ehime University, Toon, Japan; 5 Department of Information Communication Technology ICT Division, Government of Bangladesh, Dhaka, Bangladesh; 6 Department of Computer Information Science, Higher Colleges of Technology, Abu Dhabi, United Arab Emirates; 7 Department of Neurodevelopmental Disorders, Nagoya City University Graduate School of Medical Sciences, Nagoya, Japan; Federal University of Santa Maria: Universidade Federal de Santa Maria, BRAZIL

## Abstract

**Aims:**

Accurate electrocardiogram (ECG) interpretation is essential for appropriate clinical decision-making, yet competency remains suboptimal among many healthcare professionals. Although computerized adaptive testing (CAT) has the potential to provide rapid, individualized, and standardized assessment of ECG interpretation skills, its implementation requires a well-calibrated item bank based on item response theory. This study aimed to establish item parameters, evaluate the latent structure underlying ECG interpretation competency using multidimensional item response theory (MIRT), and develop an initial CAT prototype for ECG competency assessment.

**Methods and results:**

We analyzed responses from 535 healthcare professionals who completed a 50-item online ECG test previously developed and administered via an online platform. Participants represented diverse clinical roles and experience levels. Dimensionality was examined using exploratory and confirmatory MIRT analyses. A unidimensional model and a two-factor model both emerged as plausible latent structures. Item parameters, including discrimination and difficulty, were estimated from item characteristic curves. Of 50 items, 47 demonstrated strong discrimination, and difficulty parameters were appropriately distributed. Model fit analyses demonstrated that the selected model adequately represented the response data and provided a suitable basis for CAT development.

**Conclusions:**

Although both unidimensional and two-factor models demonstrated acceptable statistical performance, the unidimensional model was selected as the initial operational framework because of its simplicity and suitability for computerized adaptive testing. The calibrated ECG item bank enabled development of a preliminary CAT prototype. These findings support the feasibility of CAT as an efficient and standardized approach for assessing ECG interpretation competency across healthcare professionals. Further studies are warranted to validate its performance and educational utility in broader clinical settings.

## Introduction

Accurate electrocardiogram (ECG) interpretation is a fundamental clinical competency for healthcare professionals because it directly influences the diagnosis, risk stratification, and management of cardiovascular disease. Despite its importance, there is considerable variability in ECG interpretation skills among healthcare providers, which may contribute to diagnostic errors, delayed treatment, and adverse patient outcomes [1–3]. Recent studies have also highlighted persistent gaps in ECG interpretation competency despite ongoing educational efforts [4]. Although various educational approaches have been proposed to improve ECG interpretation skills, no consensus has been reached regarding the most effective educational strategy [5]. Consequently, the development of standardized, objective, and efficient methods for assessing ECG interpretation competency remains an important challenge in medical education.

To address this challenge, we previously initiated a multi-phase project to develop a standardized assessment tool for ECG interpretation competency. In the first phase, a 50-item multiple-choice question (MCQ) examination was developed using the RAND/UCLA appropriateness method, with item selection performed by an expert panel [6,7]. In the second phase, which is described in the present study, we applied multidimensional item response theory (MIRT) to analyze responses to a 50-item ECG MCQ test [8].

To the best of our knowledge, the application of MIRT-based approaches to ECG interpretation assessment remains limited. Furthermore, it remains unclear whether commonly used physiological or clinical classifications reflect the latent structure underlying ECG interpretation ability [8,9]. MIRT enables objective estimation of item characteristics, including discrimination, difficulty, and latent dimensions, providing the foundation for developing reliable assessment tools. Conventional fixed-form examinations require all examinees to answer the same set of questions regardless of their level of competency, which may increase testing burden while providing limited measurement efficiency. In contrast, computerized adaptive testing (CAT), which dynamically selects test items based on an individual’s ability level, has emerged as an efficient approach to assessment. By utilizing item parameters estimated through item response theory, CAT can achieve accurate and individualized assessment while requiring fewer questions than conventional fixed-form examinations [10–12]. Such an approach has the potential to reduce testing burden while maintaining measurement accuracy, making it particularly attractive for competency-based education across diverse healthcare professions.

Accordingly, the present study had two primary objectives. First, we sought to establish a methodology for estimating item parameters and identifying the latent structure underlying ECG MCQs using MIRT. Second, we aimed to develop an initial CAT prototype based on these parameters as a foundation for efficient and standardized assessment of ECG interpretation competency.

## Methods

### Study design and setting

**Fig 1:**
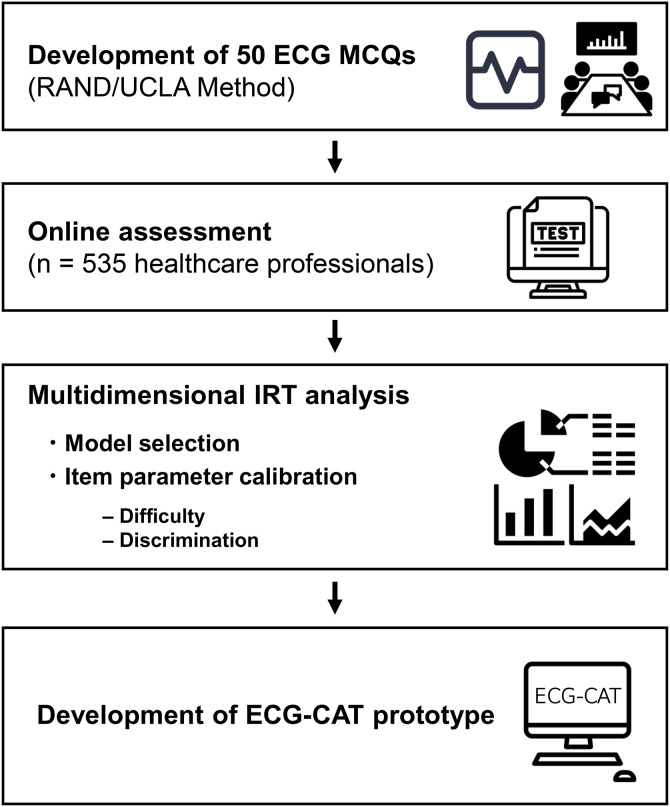
Overview of the study workflow. Fifty ECG MCQs were developed through expert consensus using the RAND/UCLA Appropriateness Method. An online assessment was subsequently conducted among 535 healthcare professionals. The collected responses were analyzed using MIRT to select the optimal model and calibrate item parameters, including difficulty and discrimination. These parameter estimates were then used to develop the ECG-CAT prototype.

The overall study workflow is illustrated in Fig 1. A detailed description of the study design has been published elsewhere; here, we provide a brief summary [8]. We conducted a cross-sectional, web-based study to evaluate ECG interpretation competency and to establish the item parameters required for CAT using a MIRT framework. We developed a dedicated online platform for this study, which enabled participants to complete the assessment remotely. The recruitment period for this study was from April 26 to July 15, 2024, and we included only participants who completed all assessment items. This study was undertaken in accordance with the Declaration of Helsinki and was approved by the Institutional Review Board of Ehime University Graduate School of Medicine (IRB number: 2209008). Written informed consent was obtained online from all participants prior to their participation and minors were not included in this study.

Details of the participant recruitment process, ECG test development, and assessment procedures are provided in the Supplemental Detailed Methods and Fig S1 in S1 File.

### Statistical analysis

We previously described the statistical methodology in detail in our study protocol [8]. Here, we briefly summarize the analytical procedures used to estimate item parameters, identify the underlying structure of ECG interpretation competency, and develop the CAT prototype. The MIRT analyses were performed to identify the underlying structure of ECG interpretation competency and to estimate the item parameters required for CAT development.

#### Sample size.

Based on prior research and simulation studies [8], we determined that a minimum of 350 participants was required to achieve stable parameter estimation for the IRT analysis. To account for an anticipated 20% dropout or incomplete response rate, we aimed to recruit 438 participants to ensure at least 350 complete datasets.

#### Model selection, estimation, and fit.

We first explored the dimensionality of the data using exploratory factor analysis (EFA). Based on the EFA results, we specified several candidate models and assessed their fit through confirmatory factor analysis (CFA) within a MIRT framework. Specifically, we employed a bifactor MIRT model to account for both general and domain-specific factors, evaluating model fit using standard indices. The exploratory IRT model included: (1) transformation of dichotomous responses via quasi-polychoric correlations; (2) parameter estimation using the Metropolis-Hastings Robbins-Monro algorithm; (3) oblique (geomin) factor rotation; and (4) retention of factors with loadings ≥ 0.40 [13–15]. Competing models with varying dimensional structures were compared based on –2 log likelihood (–2LL), Akaike Information Criterion (AIC), Bayesian Information Criterion (BIC), and root mean square error of approximation (RMSEA), with RMSEA < 0.08 considered acceptable [16–18]. Local dependence among items was examined as part of the IRT assumption checks, with Cramer’s V < 0.2 indicating acceptable levels of dependence [19]. Based on the exploratory factor analysis results, we estimated both unidimensional and bifactor models using confirmatory factor analysis within a three-parameter logistic (3PL) MIRT framework. Because all items were five-option multiple-choice questions, the guessing parameter was initialized at 0.20 in the mirt model. This value was used only as a starting value for estimation and was not fixed. Accordingly, item-specific guessing parameters were estimated and allowed to vary across items. Parameter estimation was performed using the Gauss-Hermite quadrature method within the Expectation-Maximization (EM) algorithm [20]. Model fit was evaluated using the previously described indices (–2LL, AIC, BIC, and RMSEA). Based on the selected best-fitting model, we proceeded to estimate both item and person parameters.

#### Test, item, and person diagnostics.

We evaluated item fit using Zh statistics, where values greater than zero indicated better-than-expected fit, and values less than zero indicated worse-than-expected fit [21]. We reported item discrimination and intercept parameters, along with multivariate discrimination and intercepts, to assess the utility and performance of individual items [22].

#### Latent trait (θ) estimation.

We estimated the latent trait (θ) for each respondent using maximum a posteriori (MAP) estimation, a Bayesian approach that incorporates a normal prior distribution (mean = 0, standard deviation = 1) [23]. This method allowed us to obtain stable person-level ability estimates, accounting for both observed responses and prior expectations.

#### Development of a CAT prototype.

Based on the item parameters estimated from the MIRT analysis, we developed a prototype CAT system designed to estimate ECG interpretation competency efficiently by dynamically selecting items according to each examinee’s estimated ability. The prototype included the following components: (1) a login page requiring an ID and password, (2) an introductory page explaining the system’s objective, (3) a demographic data entry page, (4) a technical information page outlining the test procedure, (5) adaptive test pages, where items were dynamically selected based on predefined parameters to converge toward the test target. The number of administered questions varied according to each respondent’s answer pattern, and (6) a results page displaying the estimated latent trait, its position within the theoretical distribution, and the respondent’s response pattern.

#### Statistical software.

All analyses were conducted using R (version 4.2.21; R Foundation for Statistical Computing, Vienna, Austria). The ‘mirt’ package was used for MIRT analysis [20], and the ‘mirtCAT’ package was employed for CAT development [24].

## Results

### Participants and test completion

Between April 26 and July 21, 2024, we registered 653 participants after manually screening them according to the inclusion and exclusion criteria. Among these, 571 participants logged into the system and answered at least one question. A total of 535 participants completed all 50 items during the study period and were included in the final analysis. Supplemental Fig S2 in S1 File presents the distribution of correct response rates for both items and participants. The item-level correct response rates were approximately uniformly distributed between 0.2 and 0.8, suggesting a balanced difficulty level across items. In contrast, participant scores followed an approximately normal distribution with a slight left skew and a mode around 20 correct responses.

### Dimensionality analysis: Exploratory and confirmatory IRT

**Fig 2:**
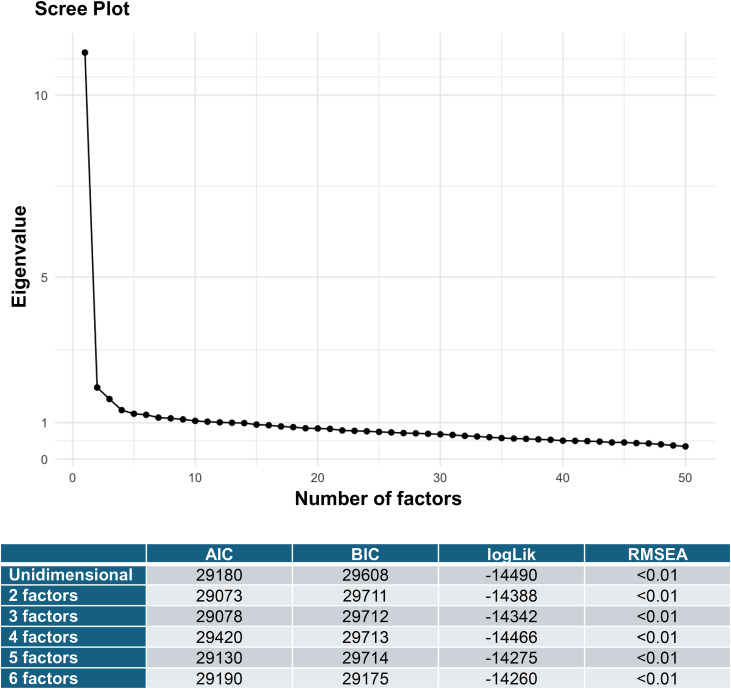
Scree plot of eigenvalues, along with AIC, BIC, and −2 log-likelihood values for each model. The first eigenvalue is substantially larger than the subsequent eigenvalues.

We began by evaluating the dimensionality of the assessment using exploratory IRT factor analysis. We compared model fit indices across models ranging from one (unidimensional) to six latent factors. Fig 2 presents the results of the exploratory IRT factor analysis, including the scree plot of eigenvalues, alongside AIC, BIC, and –2 log-likelihood (–2LL) values for each model. These indices were used for initial exploration of dimensionality and are not directly comparable to the confirmatory model fit indices presented in Table 2. The first eigenvalue was substantially larger than the subsequent ones, indicating a dominant underlying factor and limited contribution from additional dimensions.

**Table 1:** Comparison of loading values for each question between unidimensional and bifactor models.

	*Unidimensional*	*Bifactor*								
		2 factors			5 factors					
*Q number*	*F1*	*G*	*S1*	*S2*	*G*	*S1*	*S2*	*S3*	*S4*	*S5*
1	0.84	0.77	0.20		0.81			−0.56		
2	0.64	0.64	0.15		0.64				0.07	
3	0.79	0.79	0.13		0.82	−0.12				
4	0.79	0.78	0.12		0.77		0.50			
5	0.91	0.92	0.02		0.88		0.32			
6	0.69	0.72		0.44	0.73					0.41
7	0.87	0.91	−0.11		0.88					
8	0.85	0.76	0.50		0.85		0.13			
9	0.58	0.57		0.04	0.59	−0.04				
10	0.73	0.69	0.19		0.77		0.40			
11	0.79	0.73	0.24		0.79			−0.17		
12	0.83	0.80	0.18		0.84					−0.12
13	0.81	0.75	0.21		0.83				−0.23	
14	0.80	0.77	0.20		0.80		0.22			
15	0.93	0.90	0.25		0.93		0.03			
16	0.84	0.72	0.40		0.83		0.04			
17	0.64	0.64		0.26	0.63	0.35				
18	0.83	0.86	−0.08		0.82		0.12			
19	0.57	0.58	−0.04		0.62				0.71	
20	0.54	0.47		0.40	0.52					0.55
21	0.87	0.82	0.28		0.88	−0.08				
22	0.79	0.78	0.01		0.79		−0.14			
23	0.83	0.80		0.41	0.82					0.28
24	0.82	0.84	−0.08		0.82			0.21		
25	0.73	0.72		0.35	0.70					0.52
26	0.91	0.99	−0.12		0.92		0.19			
27	0.77	0.78		0.02	0.76			0.49		
28	0.67	0.69	−0.08		0.66		0.05			
29	0.86	0.79		0.59	0.76	0.64				
30	0.88	0.87	0.11		0.88					0.17
31	0.76	0.76	−0.01		0.76					−0.05
32	0.79	0.77		0.15	0.79			−0.01		
33	0.40	0.40			0.39		0.11			
34	0.74	0.56	0.67		0.79					−0.43
35	0.90	0.84	0.36		0.90	−0.16				
36	0.55	0.52	0.12		0.52		0.35			
37	0.51	0.53	−0.15		0.51	0.27				
38	0.62	0.64	−0.09		0.69	−0.16				
39	0.77	0.73	0.17		0.77		0.08			
40	0.64	0.61	0.39		0.71					−0.43
41	0.88	0.83	0.32		0.88			0.04		
42	0.86	0.84		0.28	0.86	0.24				
43	0.47	0.50		0.40	0.46				0.18	
44	0.73	0.70	0.18		0.74		−0.10			
45	0.77	0.74	0.20		0.76					0.01
46	0.93	0.89	0.22		0.92					−0.08
47	0.87	0.81	0.23		0.85		0.15			
48	0.76	0.72		0.27	0.70	0.57				
49	0.67	0.63		0.51	0.66			0.21		
50	0.14	0.16	−0.14		0.31			0.34		

**Table 2:** Comparison of AIC, BIC, and Log-Likelihood for each model.

	AIC	BIC	logLik	X2	df	p
**Unidimensional**	29077	29719	−14388			
Bifactor (2 factors*)	28966	29823	−14283	211	50	0
Bifactor (5 factors**)	29035	29892	−14318	not interpretable	0	

*2 factors = disease having specific ECG findings (yes/no), **5 factors = physiological categories (ischemia/infarction, rhythms, structural, metabolic/inflammatory and others)

Next, we conducted confirmatory factor analysis to evaluate these candidate models within a MIRT framework. Specifically, we tested a unidimensional model and a two-dimensional bifactor model under the hypothesis that disease-specific ECG findings might correspond to latent constructs. Additionally, we tested a five-dimensional bifactor model, reflecting our initial assumption that items might cluster according to physiological categories (ischemia/infarction, rhythm disorders, structural abnormalities, metabolic/inflammatory conditions, and others).

Table 1 shows the item loading values for the unidimensional and bifactor models. Loadings for the unidimensional model and the general factor of the bifactor model were similar. However, the loadings for the five specific factors in the bifactor model were generally low and sometimes negative, indicating poor alignment with the proposed dimensions. Table 2 presents AIC, BIC, and log-likelihood values for all three models derived from confirmatory factor analysis within the MIRT framework. While the two-factor model had the lowest AIC, the unidimensional model yielded the smallest BIC and log-likelihood and showed more stable item loadings across the test. In contrast, both bifactor models demonstrated instability due to high variation in item loadings. Model-comparison indices suggested that both unidimensional and bifactor two-factor solutions were plausible. The bifactor two-factor model showed somewhat better AIC and log-likelihood values, whereas the unidimensional model was favored by BIC. Because the primary purpose of the present study was to establish an initial, operationally simple framework for CAT prototype development, the unidimensional model was adopted for subsequent item calibration and CAT implementation. The chi-square statistic for the bifactor five-factor model was not interpretable for formal statistical comparison because the model yielded 0 degrees of freedom, suggesting an unstable or misidentified solution in the present sample. Accordingly, this model was not used as a basis for inferential model selection.

### Item parameter estimation and fit using the unidimensional IRT model

**Fig 3:**
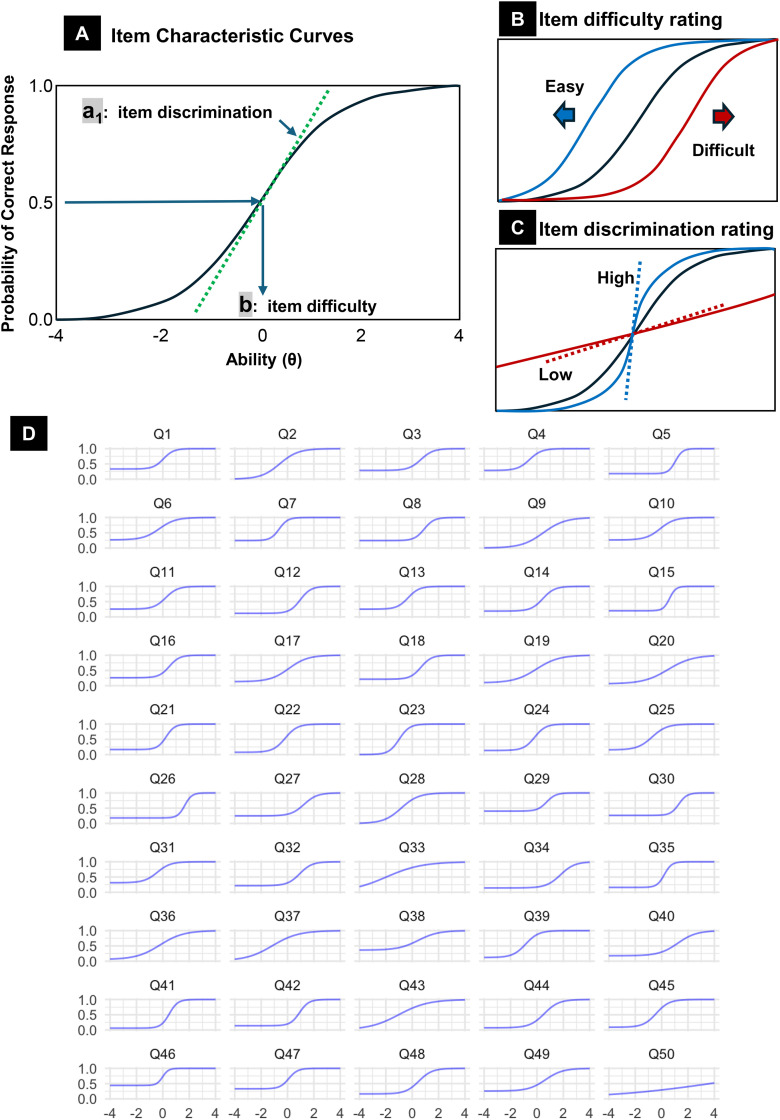
Item characteristic curve (ICC) analysis for identifying high-quality questions and determining difficulty levels. Panels A, B, and C show a description (A) and visual evaluations (B and C) of ICCs. The vertical axis represents the probability of a correct response, while the horizontal axis represents ability (θ). Item discrimination (a₁) is reflected in the slope of the curve, and item difficulty (b) is represented by the ability value (θ) at which the probability of a correct response is 0.5 (A). The ICC shifts to the left for easier questions (blue line) and to the right for more difficult questions (red line) (B). Questions with higher discrimination have steeper slopes (blue line), whereas questions with lower discrimination have shallower slopes (red line) (C). Panel D shows ICCs for each question. Questions Q33, Q43, and Q50 exhibit gentle slopes, indicating relatively low discrimination. In contrast, Q23, Q35, and Q26 show good discrimination, with Q23 being the most difficult, followed by Q35 and Q26. These curves illustrate how CAT uses item difficulty and discrimination to select the most informative question according to the examinee’s estimated ability.

This analysis was performed to evaluate whether the ECG item bank was suitable for CAT. Specifically, we examined whether the questions covered an appropriate range of difficulty and could effectively distinguish between participants with different levels of ECG interpretation competency. We estimated item parameters under the unidimensional 3PL IRT model, as this model best fit the data. Fig 3 and Supplemental table S1 in S1 File present item discrimination (a_1_) and difficulty (b) parameters alongside item characteristic curves (ICCs) for each question. The item discrimination parameter exceeded 1.0 in 47 of 50 items, indicating good ability discrimination, although a few items showed lower discrimination reflected in their ICCs. These results demonstrate that the unidimensional IRT model successfully extracted high-quality items with strong discriminative power. The distribution of item difficulty (mean ± SD = 0.24 ± 0.93) was approximately normal with a slight right skew. No problematic local dependence was identified according to the predefined criterion of Cramer’s V < 0.2. Overall, these findings indicate that the ECG item bank provides an appropriate range of item difficulty and discrimination to support CAT-based assessment.

### Item and person fit diagnostics

To evaluate whether the selected IRT model adequately represented the observed response patterns, we examined both item- and person-level model fit. Using Zh statistics, we evaluated item and person fit. Item-level Zh values are provided in Supplementary table S3 in S1 File to improve transparency. Overall, the Zh statistics did not indicate marked item misfit, although several items with low discrimination were identified as candidates for future revision. Person-fit Zh statistics were centered around zero with a near-normal distribution, reflecting variable model fit across individuals (Supplemental Fig S3A in S1 File). These findings support the overall adequacy of the selected model for subsequent CAT development while also identifying a small number of items that may benefit from future refinement.

### Latent trait (θ) estimation and distribution

Each participant’s ECG interpretation ability was expressed as a latent trait (θ), which was subsequently used by the CAT algorithm to adaptively select test items. We estimated respondents’ latent trait scores (θ) using maximum a posteriori (MAP) estimation, which assumes a normal prior (mean = 0, SD = 1). The resulting θ estimates distributed approximately normally between –2 and 2 without apparent skewness (Supplemental Fig S3B in S1 File). This distribution indicates that the assessment was able to distinguish participants across a broad range of ECG interpretation competency.

### Correlations between item and person parameters and scores

**Fig 4:**
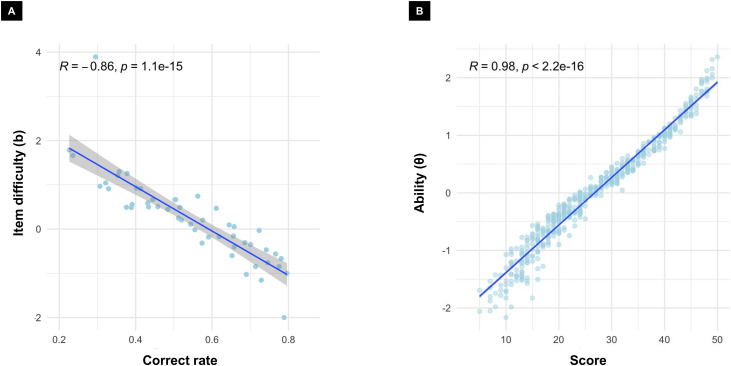
Correlations between correct answer rate and item difficulty (A), and between total score and ability (θ) (B). (A) Although two of the 50 questions were outliers, there was a strong negative correlation between the correct answer rate and item difficulty. (B) Total score and ability (θ), as estimated by MIRT, showed a strong positive correlation.

Fig 4 shows a significant negative correlation between item difficulty and correct response rate, confirming expected relationships. However, two items ([Q33] and [Q50]) deviated from this overall pattern. Additionally, participants’ total scores correlated positively with their estimated ability (θ), supporting the validity of the latent trait estimates. Together, these findings support the validity of the estimated item difficulty and participant ability and demonstrate good agreement between the IRT-based estimates and observed examination performance.

### Development of a CAT prototype

Using the parameter estimates from the unidimensional IRT model, we developed an initial version of the computerized adaptive testing (CAT) prototype. The purpose of this prototype was to demonstrate how the estimated item parameters could be applied to provide an individualized assessment while reducing the number of questions administered. The item selection algorithm was configured with the following settings:

- **Adaptive criterion:** Maximum information
- **First item:** Randomly selected from items with difficulty parameters between –0.2 and 0.2
- **Stopping rules:**
  - Minimum standard error of estimation: 0.3
  - Maximum change in latent trait estimate between items: 0.025
  - Maximum number of administered questions: 50

These parameters were provisional and intended for further refinement in subsequent research.

The developed CAT prototype operates as follows after participants log into the system. An example is shown in Supplemental Fig S4 in S1 File. Following a brief introduction, participants enter their personal information, after which an ECG test question is presented. Each question includes a textual description, an ECG image, and five multiple-choice options. The first question is selected at random. Subsequently, the difficulty of each subsequent question is automatically adjusted based on the participant’s ECG interpretation ability. The number of administered questions varies according to the examinee’s responses. Upon completing the test, participants can immediately view their ability estimate (theta), review the progression of their assessment, and download a report. In the example provided, ECG interpretation skill was determined in less than six minutes using only 14 questions. This example demonstrates the feasibility of using CAT to efficiently assess ECG interpretation competency while substantially reducing the number of administered questions.

## Discussion

An evaluation using MIRT on the ECG online test results, developed through the RAND/UCLA method in a previous study, revealed the following: (1) Both unidimensional and two-factor models emerged as potential latent structures; however, when including the physiological five-factor model, the unidimensional model selected as the initial operation model. (2) Item discrimination parameters confirmed that the test set had high discriminatory power. (3) Item difficulty for each question was clearly estimated using MIRT. Based on these findings, a CAT prototype based on the unidimensional model was successfully developed. This prototype demonstrates how MIRT-derived item parameters can be translated into an adaptive assessment system that provides individualized evaluation with fewer test items. To our knowledge, this represents the first ECG interpretation assessment test constructed using MIRT-based CAT.

### MIRT-based evaluation of potential latent factors for ECG MCQs

An evaluation using MIRT on the ECG online test results revealed that both unidimensional and two-factor models emerged as statistically plausible latent structures. However, although the two-factor model showed slightly improved fit on some statistical indices, descriptive examination did not support a clinically meaningful or practically useful separation of ECG competency domains. Therefore, for the purpose of initial CAT prototype development, we adopted the unidimensional model as a more parsimonious and interpretable operational framework. These findings suggest that, at least for the present item bank, ECG interpretation competency may be operationalized as a largely integrated latent trait for practical assessment purposes.

ECG abnormalities are typically classified into two categories: waveform-based classification and clinical diagnosis such as pathophysiology of the underlying disease. One of the most widely used waveform-based systems is the Minnesota Code, developed by Blackburn et al. at the University of Minnesota [25]. However, the Minnesota Code is not a standard of clinical practice; rather, it is used as an objective indicator in epidemiological studies, clinical research and health examinations. Therefore, classification based on clinical diagnosis may be more suitable for enhancing ECG interpretation skills in daily clinical practice. However, to date, no systematic studies have examined the potential latent factors underlying ECG interpretation [8,9]. In this study, MIRT suggested that a unidimensional model was the most practical operational framework for the present item bank, although a two-factor solution was also statistically plausible. Importantly, the poorer fit of models based on predefined physiological categories does not necessarily mean that ECG interpretation lacks multidimensional aspects. Rather, our findings suggest that, in this dataset, the predefined physiological classification was not strongly supported as the latent structure underlying item responses. This may reflect the uneven distribution of items across categories, the overlap of cognitive processes required across ECG diagnoses, or the possibility that ECG interpretation functions as a more integrated, non-fragmented competency in practical assessment settings. The probability of guessing differed across items, as expected, and was estimated rather than fixed in the final model. This approach better reflects the variability in item characteristics compared to assuming a constant guessing parameter. Taken together, these findings support the use of a unidimensional framework for the initial implementation of CAT, while acknowledging that ECG interpretation remains a complex competency that may include multidimensional components.

### Impact of MIRT-based Item discrimination on creating quality questions

MCQs are widely used in learning and teaching across a variety of disciplines, not only in medicine. However, item-writing flaws in MCQs have been reported to be highly prevalent [6]. A survey of MCQs in published books found that only 23% were free from item-writing flaws [26]. Since flawed MCQs in a test set are known to reduce learning efficiency, improving the quality of MCQs is essential [27]. We previously reported that item selection by experts with a consensus-building method, RAND/UCLA can contribute to creating higher-quality tests [6]. The present study utilized a validated 50-question test set developed using this methodology. The MIRT-based assessment of item identification confirmed that the test set had high discriminatory power overall; however, 3 out of the 50 questions demonstrated poor discrimination. Moreover, 2 of these 3 questions exhibited extreme variation in item difficulty. These findings suggest that quantitative evaluation of item discrimination via MIRT, in addition to the RAND/UCLA consensus, may further enhance the quality of MCQs. CAT has the advantage of providing an accurate assessment of competence using fewer questions than classical examinations. On the other hand, CAT has the disadvantage that it requires a larger pool of quality questions to ensure that the questions are appropriate to the ability of the candidates [10–12]. Therefore, the results of this study will be of great help in maintaining the quality of questions in the ECG item pool to be used for CAT implementation.

Although overall item discrimination was strong, three items (Q33, Q43, and Q50) showed relatively low discrimination. These items should be considered candidates for revision or replacement during future expansion of the ECG item bank, because poorly discriminating items may reduce the precision of ability estimation in CAT-based assessment. In the present study, we retained these items because our goal was initial prototype development using the existing validated 50-item set; however, their performance will need to be reassessed in subsequent iterations. In addition, two of these items (Q33 and Q50) were also identified as outliers in the relationship between item difficulty and correct response rate (Fig 4A). Such deviations may reflect atypical ECG tracings, ambiguity in diagnostic interpretation, or item-specific features that are not fully captured by the overall difficulty parameter. These items therefore merit qualitative review in addition to psychometric evaluation and should be carefully examined during future refinement of the ECG item bank.

### Usefulness of CAT in enhancing learning efficiency

Conventional examinations based on classical test theory (CTT) rely on total and deviation scores, but they present several limitations: (1) Scores can fluctuate depending on the difficulty of the questions and the characteristics of the tested population; (2) They do not account for differences in candidate ability or individual item difficulty; (3) Scores from different test sets cannot be directly compared. In contrast, the MIRT-based CAT estimates each examinee’s ability value based on item characteristics such as question difficulty, discrimination, and examinee correctness or incorrectness on each item [8]. Thus, CAT provides a more accurate and rapid assessment of each candidate’s competence compared to traditional CTT-based exams. Moreover, CAT can reduce the burden on candidates by shortening test durations [10–12]. Furthermore, as the questions are not fixed, it allows for a fair comparison of competence no matter who takes the test or where they take it. Despite these advantages, accurate difficulty assessing the pooled questions is essential because CAT must extract questions that match the difficulty level of each examinee. This study demonstrated that MIRT enabled accurate difficulty rating for each question. From an educational perspective, CAT may allow learners to receive rapid and individualized feedback while reducing testing burden, making repeated assessment more practical during ECG training. These findings may contribute to improving the accuracy of CAT, but the feasibility and performance of the developed CAT prototype require further validation. Although the present findings support the psychometric feasibility of the CAT prototype, additional studies are needed. In particular, future studies should examine criterion-related validity by comparing CAT-based ability estimates with actual clinical performance in real-world patient-care settings.

### Prospects for the development of efficient ECG learning systems

Acquisition of ECG interpretation skills requires not only accurate assessment of the skills but also improved training methods. A variety of instructional approaches have been explored, including face-to-face teaching and e-learning formats [28]. A meta-analysis showed no significant difference in ECG competence between face-to-face and online instruction [29], while several studies have shown that blending online education with face-to-face teaching might be effective [30,31]. Furthermore, e-learning platforms and mobile applications have also demonstrated utility in ECG education [32,33]. Thus, the effectiveness of each training method varies from study to study, and the optimal training method has not yet been established. Online education offers many educational opportunities, although there is a lack of communication and limited interaction. The COVID-19 pandemic accelerated the transition from conventional face-to-face education to online formats, highlighting the need for more robust and interactive digital learning systems [34].

Therefore, the goal of our ECG project is to develop an online ECG learning platform that integrates lecture-based e-learning modules with CAT. The lecture-based e-learning modules consist of dozens of video lecture groups, with each lecture scheduled for 10–15 minutes. The effectiveness of these modules will be scientifically tested in the future. The insights throughout the project have potential applications not only in ECG education, but also in broader undergraduate medical education. Such an integrated platform may facilitate competency-based ECG education by combining adaptive assessment with targeted learning content.

## Limitation

This study has several limitations. First, the analysis was based on a relatively small set of 50 ECG MCQs. Although the results suggest a unidimensional latent structure, further validation using a larger and more diverse item pool is required. Second, participants were recruited from healthcare professionals with varying levels of ECG expertise, primarily in Japan. Although this heterogeneous sample reflects real-world educational settings, the participant composition may have influenced item parameter estimation and may limit the generalizability of the findings to other healthcare populations or international settings. Future studies including more geographically and professionally diverse populations are warranted. Third, while a prototype CAT system was developed, its effectiveness has not yet been empirically validated in real-world or clinical settings. Future studies are needed to evaluate its performance. Fourth, differential item functioning (DIF) analysis was not performed to assess whether item parameters were invariant across participant subgroups. Consequently, potential measurement bias between subgroups cannot be excluded. Future studies should evaluate DIF to confirm measurement invariance and ensure the fairness of the ECG-CAT across diverse healthcare professionals. Finally, this study focused specifically on ECG interpretation, and the generalizability of these findings to other domains of medical education remains to be determined.

## Conclusions

MIRT analysis suggested that a unidimensional model provided the plausible fit among the candidate latent factors, although this may represent a practical simplification for the initial prototype. Furthermore, the analysis enabled the precise calibration of difficulty levels for the ECG MCQs. Using the parameter estimates obtained through MIRT analysis, we successfully developed a preliminary prototype CAT system for assessing ECG interpretation skills. These findings support the feasibility of a CAT-based approach for assessing ECG interpretation skills and suggest that, in the present dataset, the predefined physiological category structure was not strongly supported as the latent basis of item responses. However, further refinement of low-discrimination items and future criterion-validity studies are needed before broader clinical implementation. If validated in larger and more diverse populations, this approach may provide an efficient and standardized method for assessing ECG interpretation competency in healthcare education.

## Supporting information

S1 File Supplemental materials. Supplemental methods, participant expertise classification, supplementary figures, item parameters, and item-fit statistics.
(DOC)
