# Supplementary material for: Development and initial validation of a computerized adaptive test prototype for ECG interpretation using item response theory
Source: PLoS One. 2026 Jul 17;21(7):e0344341. doi: 10.1371/journal.pone.0344341 (PMC13379108; doi:10.1371/journal.pone.0344341)
Supplement: S1 File — Supplemental methods, participant expertise classification, supplementary figures, item parameters, and item-fit statistics. (DOC) [file pone.0344341.s001.doc]

**SUPPLEMENTAL MATERIALS**

**Supplemental Methods**

***Participants***We targeted a diverse group of individuals likely to engage in regular ECG interpretation, including cardiologists, non-cardiology physicians, residents, medical students, nurses, nursing students, medical engineers, paramedics, and medical clerks. We recruited participants from institutions affiliated with Ehime University, such as hospitals, the School of Medicine, the School of Nursing, and local fire stations. We applied the following inclusion criteria: (1) age 18 years or older; (2) completion of a systematic course on ECG interpretation (e.g., through medical, health, or nursing schools, or postgraduate continuing education); and (3) proficiency in Japanese sufficient to understand and respond to the assessment questions. We excluded individuals who (1) had never completed a formal lecture or course on ECG interpretation or (2) lacked regular opportunities to evaluate ECGs. To encourage participation, we provided each participant with a ¥2,000 (approximately US$15) Amazon gift card.

***Development of the ECG Online Test***We previously reported the detailed methodology used to develop the 50-item ECG test.^6^ In brief, following the RAND/UCLA Appropriateness Method (RAM) manual, we convened a panel of nine experts who participated in a structured three-phase consensus process. Two experienced cardiologists initially drafted 100 ECG questions. During panel meetings, we evaluated the content validity of each item and assigned it to one of five physiological domains—ischemia/infarction, rhythm disorders, structural abnormalities, metabolic/inflammatory conditions, and others—based on expert consensus. The panel deemed 77 questions appropriate, from which we selected 50 for inclusion in the final online assessment.

***Process of Assessment***We invited interested individuals to register through a Google Form, which collected demographic and background information, including name, age, academic and job titles, prior experience with systematic ECG interpretation courses, and email address. After confirming eligibility, we issued a unique ID and password to each participant via email, granting access to the online assessment platform. The assessment consisted of 50 multiple-choice questions (MCQs), each requiring the selection of one correct answer from five options. We presented the questions in a random order. Although the original protocol specified a 90-minute time limit, we omitted this restriction and required participants to complete the assessment within the designated study period. Upon completion, participants were shown their total number of correct responses; no additional feedback was provided. An example question is shown in Supplemental figure 1.

A total of 535 participants were included in the analysis, comprising cardiologists (n=41), medical technologists (n=111), non-cardiologists (n=42), senior residents (n=13), junior residents (n=21), clinical engineers (n=31), medical students (n=77), paramedics (n=33), nurses (n=144), nursing students (n=8), and others (n=14). For descriptive purposes, participants were broadly categorized into three groups based on presumed ECG interpretation expertise: experts (e.g., cardiologists), intermediate (e.g., non-cardiologists, residents, and allied health professionals), and novices (e.g., students), (Supplementary table S1). The distribution of expertise levels was considered in the interpretation of MIRT parameter estimates.

**Supplementary table S1. Participant Expertise Levels**

| **Expertise level** | **Occupation** | **N=535** | **%** |
| --- | --- | --- | --- |
| Expert | Cardiologists | 41 | 7.7 |
| Intermediate | Non-cardiologists, residents, nurses, technologists, engineers, paramedics | 395 | 73.8 |
| Novice | Medical students, nursing students | 85 | 15.9 |
| Others/Unclassified | Others | 14 | 2.6 |

**Supplemental figures**

**Supplemental** **figure 1. An example of a multiple-choice question answered online by participants.**

A clinical question, including the patient’s age, gender, and chief complaint, is presented in the top section. Participants select one answer from five options. An electrocardiogram image is shown in the bottom section, and the correct answer to this question is "Normal sinus rhythm."


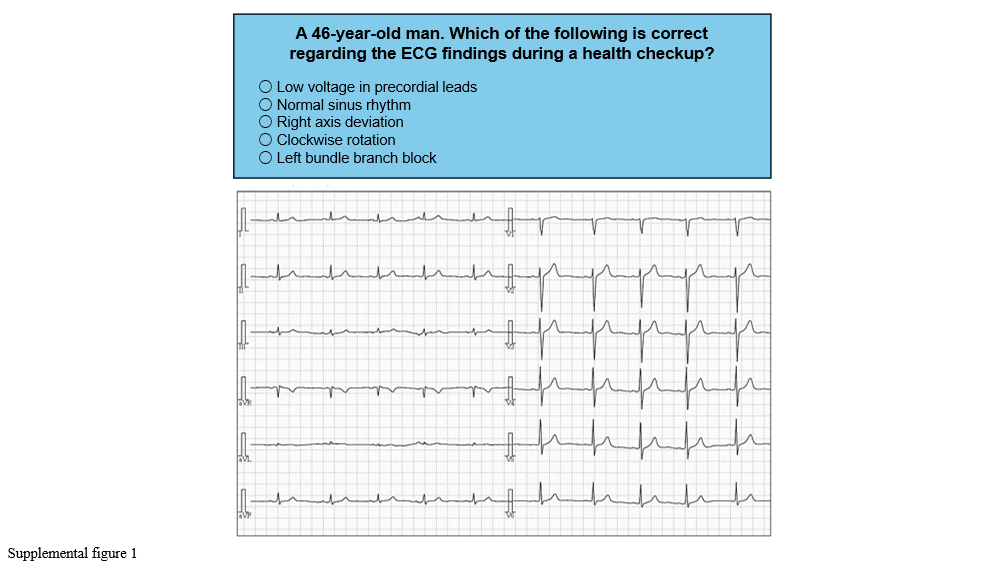


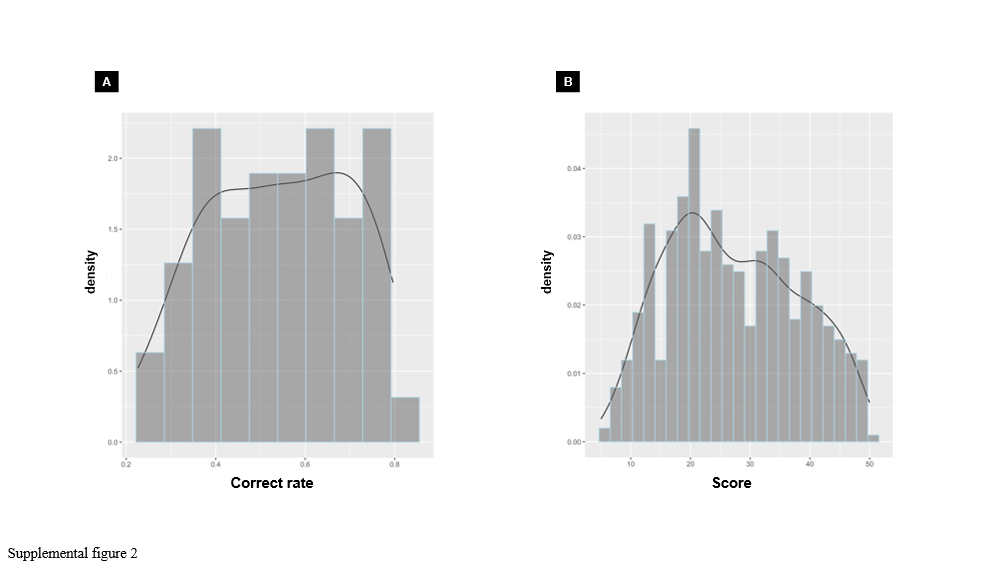
**Supplemental figure 2. Distribution of question accuracy rates (A) and participant scores (B).**

**Supplemental figure 3. (A) Assessment of item and person fit using Zh statistics, (B) Distribution of item difficulties and participant abilities (θ).**

**
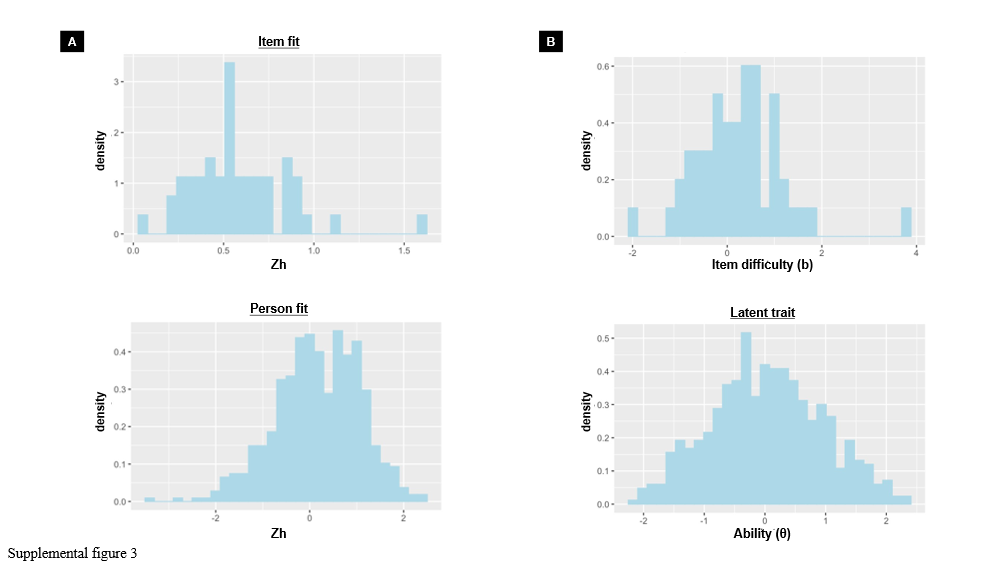
**

**Supplemental figure 4. Example of the usage flow for the Ehime ECG-CAT.**

**A:** Brief introduction to the system. **B:** Participant information entry: participants input their age, gender, occupation, and frequency of ECG interpretation. **C, D:** Notification of test results: examinees can view their estimated level of ECG interpretation ability (C) and a detailed breakdown of the decision-making process for each question (D). In this example, ability estimation was completed with only 14 questions.


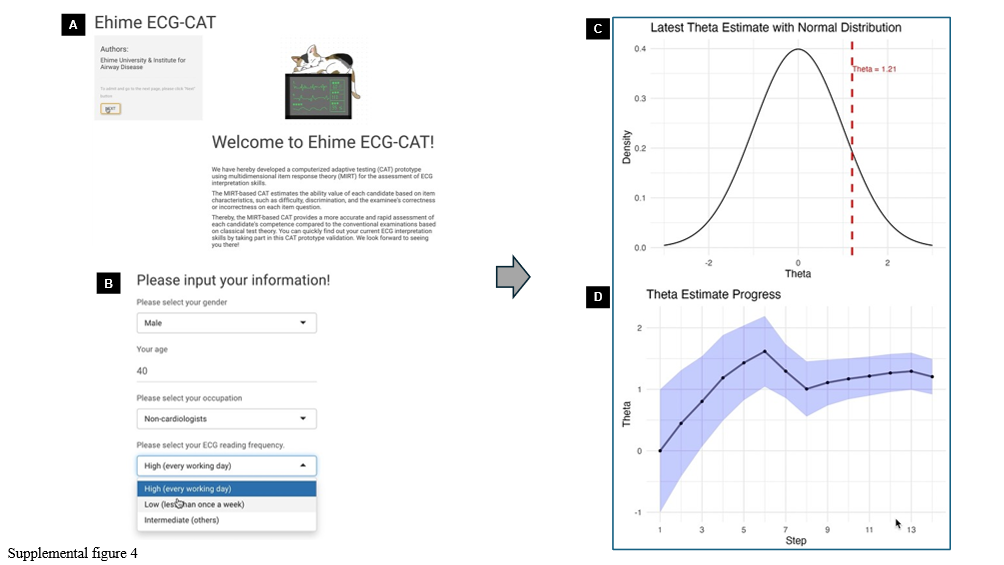


**Supplemental table S2.** Item parameters for each 50-question.

| Q number | **a_1_** | **b** | **g** |
| --- | --- | --- | --- |
| 1 | 2.6 | 0.0 | 0.3 |
| 2 | 1.4 | -0.6 | 0.0 |
| 3 | 2.2 | 0.7 | 0.3 |
| 4 | 2.2 | -0.6 | 0.3 |
| 5 | 3.7 | 1.0 | 0.2 |
| 6 | 1.6 | -0.3 | 0.3 |
| 7 | 3.1 | -0.6 | 0.2 |
| 8 | 2.7 | 0.9 | 0.2 |
| 9 | 1.2 | 0.5 | 0.0 |
| 10 | 1.8 | -0.1 | 0.3 |
| 11 | 2.2 | 0.2 | 0.3 |
| 12 | 2.5 | 1.0 | 0.1 |
| 13 | 2.4 | -0.3 | 0.3 |
| 14 | 2.3 | 0.4 | 0.2 |
| 15 | 4.2 | 0.6 | 0.2 |
| 16 | 2.6 | 0.5 | 0.3 |
| 17 | 1.4 | 0.1 | 0.1 |
| 18 | 2.5 | 0.7 | 0.2 |
| 19 | 1.2 | 0.0 | 0.1 |
| 20 | 1.1 | 0.5 | 0.1 |
| 21 | 3.1 | 0.2 | 0.2 |
| 22 | 2.2 | -0.2 | 0.1 |
| 23 | 2.6 | -1.0 | 0.0 |
| 24 | 2.4 | -0.2 | 0.1 |
| 25 | 1.8 | -0.7 | 0.2 |
| 26 | 3.8 | 1.7 | 0.2 |
| 27 | 2.0 | 1.3 | 0.2 |
| 28 | 1.5 | -0.8 | 0.0 |
| 29 | 2.9 | 0.7 | 0.4 |
| 30 | 3.2 | 1.3 | 0.3 |
| 31 | 2.0 | -0.4 | 0.3 |
| 32 | 2.2 | 0.9 | 0.2 |
| 33 | 0.7 | -2.0 | 0.0 |
| 34 | 1.9 | 1.8 | 0.1 |
| 35 | 3.6 | 0.2 | 0.2 |
| 36 | 1.1 | -0.2 | 0.1 |
| 37 | 1.0 | -1.1 | 0.0 |
| 38 | 1.3 | 0.5 | 0.4 |
| 39 | 2.1 | -0.9 | 0.1 |
| 40 | 1.4 | 1.2 | 0.2 |
| 41 | 3.1 | 0.5 | 0.1 |
| 42 | 2.9 | 0.9 | 0.1 |
| 43 | 0.9 | -1.0 | 0.0 |
| 44 | 1.8 | 0.6 | 0.1 |
| 45 | 2.0 | -0.4 | 0.1 |
| 46 | 4.5 | 0.0 | 0.4 |
| 47 | 3.0 | 0.1 | 0.3 |
| 48 | 2.0 | 0.5 | 0.2 |
| 49 | 1.5 | 0.7 | 0.3 |
| 50 | 0.2 | 3.8 | 0.0 |

**Supplemental table S3.** Item-level Zh fit statistics for the final unidimensional model.

| Q number | **Zh** |
| --- | --- |
| 1 | 0.66 |
| 2 | 0.86 |
| 3 | 0.49 |
| 4 | 0.70 |
| 5 | 0.46 |
| 6 | 0.53 |
| 7 | 1.12 |
| 8 | 0.34 |
| 9 | 0.52 |
| 10 | 0.62 |
| 11 | 0.52 |
| 12 | 0.27 |
| 13 | 0.86 |
| 14 | 0.53 |
| 15 | 0.67 |
| 16 | 0.47 |
| 17 | 0.58 |
| 18 | 0.45 |
| 19 | 0.52 |
| 20 | 0.44 |
| 21 | 0.68 |
| 22 | 0.88 |
| 23 | 1.55 |
| 24 | 0.96 |
| 25 | 0.79 |
| 26 | 0.41 |
| 27 | 0.20 |
| 28 | 0.90 |
| 29 | 0.54 |
| 30 | 0.36 |
| 31 | 0.64 |
| 32 | 0.39 |
| 33 | 0.23 |
| 34 | 0.19 |
| 35 | 0.77 |
| 36 | 0.68 |
| 37 | 0.52 |
| 38 | 0.34 |
| 39 | 0.90 |
| 40 | 0.25 |
| 41 | 0.61 |
| 42 | 0.43 |
| 43 | 0.50 |
| 44 | 0.32 |
| 45 | 0.86 |
| 46 | 0.92 |
| 47 | 0.75 |
| 48 | 0.56 |
| 49 | 0.37 |
| 50 | 0.03 |
