# Supplementary material for: Development and initial validation of a computerized adaptive test prototype for ECG interpretation using item response theory
Source: PLoS One. 2026 Jul 17;21(7):e0344341. doi: 10.1371/journal.pone.0344341 (PMC13379108; doi:10.1371/journal.pone.0344341)
Supplement: Attachment — Submitted filename: Respnse to Reviewers_PlosOne_0423.docx [file pone.0344341.s003.docx]

April 23, 2026

**Helen Howard, MD**

Staff Editor

PLOS One

RE: PONE-D-25-57504

**“Development of a Computerized Adaptive Test for ECG Interpretation Using Item Response Theory”**

Dear Dr. Howard,

Thank you for the review of our manuscript. We have responded to comments from the Reviewer in the revised manuscript as follows:

**Reviewer #1**

**I have carefully read the full manuscript, and I must acknowledge that it is a study with genuine scientific merit and practical relevance in the field of medical education and electrocardiography. The application of MIRT and the development of a CAT prototype represent an innovative approach that can contribute meaningfully to the standardization of ECG competency assessment. However, there are several points to which the authors should pay attention:**

**Response:** We sincerely thank Reviewer #1 for the careful evaluation of our manuscript and for the insightful and constructive comments. We have revised the manuscript accordingly.

**1. The title adequately reflects the content of the study, but it could benefit from greater methodological precision. It is recommended to specify that this is an initial validation study or a development phase, to prevent readers from assuming that the CAT is already fully validated and operational.**

**Response:** We thank the reviewer for this important suggestion. We agree that the title should more clearly reflect the developmental nature of this study to avoid any misunderstanding that the CAT system is fully validated.

In response, we have revised the title to better emphasize that this study represents an initial development and validation phase. The revised title is as follows:

**“Development and Initial Validation of a Computerized Adaptive Test Prototype for ECG Interpretation Using Item Response Theory”**

**2. The introduction provides a clear context for the clinical relevance of the study, but there is some redundancy in the explanation of MIRT and CAT. It is recommended to simplify and avoid repetition to improve the overall flow. The statement that “no MIRT tools for ECG exist” should be nuanced with broader references, since there are previous approaches in medical education which, although not identical, could be considered as antecedents.**

**Response:** We thank the reviewer for this helpful comment. We have revised the Introduction to reduce redundancy, particularly in the descriptions of MIRT and CAT, and to improve overall clarity. We also incorporated the suggested references to better contextualize the current challenges in ECG education and assessment.

**3. While the Supplemental Methods provide a clearer description of the participant profiles and the recruitment process at Ehime University, the manuscript would still benefit from a detailed demographic table. Specifically, the exact proportion of experts versus novices should be reported, as a skewed distribution in clinical expertise can significantly influence the stability of the a and b parameters during MIRT calibration.**

**Response:** We thank the reviewer for this important suggestion. We have revised the manuscript to include a detailed description of participant demographics in the text, including their professional backgrounds.

In addition, we categorized participants according to their level of ECG expertise (e.g., experts, intermediate, and novices) and clarified their proportions in the revised manuscript.

We also acknowledge that the distribution of clinical expertise may influence the stability of item parameter estimates in MIRT and have added this point to the Discussion as a limitation.

**Supplemental materials:** A total of 535 participants were included in the analysis, comprising cardiologists (n=41), medical technologists (n=111), non-cardiologists (n=42), senior residents (n=13), junior residents (n=21), clinical engineers (n=31), medical students (n=77), paramedics (n=33), nurses (n=144), nursing students (n=8), and others (n=14). For descriptive purposes, participants were broadly categorized into three groups based on presumed ECG interpretation expertise: experts (e.g., cardiologists), intermediate (e.g., non-cardiologists, residents, and allied health professionals), and novices (e.g., students), (Supplementary table S1). The distribution of expertise levels was considered in the interpretation of MIRT parameter estimates.

**Supplementary table S1.** Participant Expertise Levels

| **Expertise level** | **Occupation** | **N=535** | **%** |
| --- | --- | --- | --- |
| Expert | Cardiologists | 41 | 7.7 |
| Intermediate | Non-cardiologists, residents, nurses, technologists, engineers, paramedics | 395 | 73.8 |
| Novice | Medical students, nursing students | 85 | 15.9 |
| Others/Unclassified | Others | 14 | 2.6 |

**Limitation**

The heterogeneous distribution of participant expertise may have influenced item parameter estimation, which should be considered when interpreting the results.

**4. The decision to fix the guessing parameter (c) at 0.2 requires a stronger justification. In practice, the probability of guessing varies considerably depending on the ECG pattern—for example, identifying a Wolf-Parkinson-White tracing is not comparable to recognizing a simple sinus rhythm. Fixing cat a constant value may oversimplify the reality of visual interpretation. The authors should clarify why 0.2 was chosen, whether sensitivity analyses with alternative values were performed, and discuss whether an estimated parameter or a 2PL model might have provided a more realistic representation of the data.**

**Response:** We thank the reviewer for this important comment. We agree that the probability of guessing is unlikely to be identical across ECG items. Upon revisiting our analysis, we realized that the manuscript wording was imprecise. In the mirt analysis, the value of 0.20 was used as an initial value based on the five-option multiple-choice format, but it was not fixed during estimation. The guessing parameter was estimated for each item, which is why the g values vary across items in Supplementary Table 2. We have revised the Methods and related text to clarify this point and to avoid the incorrect impression that the guessing parameter was fixed at 0.20 for all items.

**Methods:** Based on the exploratory factor analysis results, we estimated both unidimensional and bifactor models using confirmatory factor analysis within a three-parameter logistic (3PL) MIRT framework~~, fixing the guessing parameter at 0.2 to suit the binary response format~~. Because all items were five-option multiple-choice questions, the guessing parameter was initialized at 0.20 in the mirt model. This value was used only as a starting value for estimation and was not fixed. Accordingly, item-specific guessing parameters were estimated and allowed to vary across items.

**Discussion:** The probability of guessing differed across items, as expected, and was estimated rather than fixed in the final model. This approach better reflects the variability in item characteristics compared to assuming a constant guessing parameter.

**5. A key assumption of IRT is that success on one item should not condition success on another. Since in an ECG one finding (e.g., peaked T waves) is often linked to another (e.g., QRS widening in hyperkalemia), the authors should confirm whether they performed local dependence tests (such as Yen’s Q3) to validate this assumption.**

**Response:** We thank the reviewer for this important comment. We agree that local independence is a key assumption in IRT and that some ECG items may involve clinically related findings. In our analysis, local dependence among items was examined, with Cramer’s V < 0.2 indicating acceptable levels of dependence. We have clarified this point in the revised Methods section to make the assessment of local dependence more explicit.

**Methods:** Criterion (AIC), Bayesian Information Criterion (BIC), and root mean square error of approximation (RMSEA), with RMSEA < 0.08 considered acceptable.^16-18^ Local dependence among items was examined as part of the IRT assumption checks, with Cramer’s V < 0.2 indicating acceptable levels of dependence.^19^

**Results:** No problematic local dependence was identified according to the predefined criterion of Cramer’s V < 0.2.

**6. The reporting of model fit indices in Table 2 presents two critical inconsistencies. First, while the bifactor (2-factor) model shows superior fit according to AIC (28966 vs. 29077) and log-likelihood (–14283 vs. –14388), the unidimensional model was selected based primarily on BIC (29719 vs. 29823). From a methodological standpoint, AIC indicates that a specific factor structure may better explain the variance, and the authors should discuss why parsimony was prioritized over improved fit, especially given that ECG interpretation is clinically multidimensional. Second, the 5-factor bifactor model reports a chi-square value of –69 with 0 degrees of freedom, which is mathematically invalid and suggests either non-convergence of the estimation algorithm or an over-identified (saturated) model. This invalidates direct statistical comparison using chi-square tests. The authors should clarify whether the 5-factor model was discarded due to convergence issues or insufficient sample size (n = 535), and it is recommended to report this model as “non-convergent” or “misidentified” rather than presenting fit statistics that lack mathematical validity.**

**Response:** We thank the reviewer for this important comment. We agree that the two-factor model showed somewhat better fit according to AIC and log-likelihood, whereas the unidimensional model was favored by BIC. We have therefore revised the manuscript to clarify that the selection of the unidimensional model was not based on a single fit index alone. Rather, both unidimensional and two-factor structures were considered plausible from a statistical standpoint. However, after descriptively examining the two-factor solution, we found that it did not yield a clinically meaningful or sufficiently interpretable separation of latent domains for ECG competency assessment. Because the primary aim of this study was to develop an initial and practically implementable CAT prototype, we prioritized parsimony and interpretability and therefore adopted the unidimensional model as the initial operational model. We have revised the Abstract, Results, Discussion, and Conclusion accordingly.

Regarding the five-factor bifactor model, we agree that the reported chi-square value with 0 degrees of freedom is not interpretable for formal model comparison. We have therefore revised Table 2 and the related text to clarify that this model was not suitable for inferential chi-square comparison and should be interpreted cautiously as an unstable/misidentified solution rather than as evidence supporting or refuting that factor structure.

**Abstract:** A unidimensional model and a two-factor model both emerged as plausible latent structures.

Although MIRT analysis suggested that both unidimensional and two-factor models were plausible, and the unidimensional model was adopted as a practical and interpretable solution for the initial CAT prototype.

**Results:** ~~Based on these results, we selected the unidimensional and two-factor models as candidates for further analysis.~~ Model-comparison indices suggested that both unidimensional and bifactor two-factor solutions were plausible. The bifactor two-factor model showed somewhat better AIC and log-likelihood values, whereas the unidimensional model was favored by BIC. Because the primary purpose of the present study was to establish an initial, operationally simple framework for CAT prototype development, the unidimensional model was adopted for subsequent item calibration and CAT implementation.

The chi-square statistic for the bifactor five-factor model was not interpretable for formal statistical comparison because the model yielded 0 degrees of freedom, suggesting an unstable or misidentified solution in the present sample. Accordingly, this model was not used as a basis for inferential model selection.

**Discussion:** An evaluation using MIRT on the ECG online test results revealed that both unidimensional and two-factor models emerged as statistically plausible latent structures. However, although the two-factor model showed slightly improved fit on some statistical indices, descriptive examination did not support a clinically meaningful or practically useful separation of ECG competency domains. Therefore, for the purpose of initial CAT prototype development, we adopted the unidimensional model as a more parsimonious and interpretable operational framework. These findings suggest that, at least for the present item bank, ECG interpretation competency may be operationalized as a largely integrated latent trait for practical assessment purposes.

**Conclusions:** MIRT analysis suggested that a unidimensional model provided the plausible fit among the candidate latent factors, although this may represent a practical simplification for the initial prototype. Furthermore, the analysis enabled the precise calibration of difficulty levels for the ECG MCQs. Using the parameter estimates obtained through MIRT analysis, we successfully developed a preliminary prototype CAT system for assessing ECG interpretation skills. These findings support the feasibility of a CAT-based approach for assessing ECG interpretation skills and suggest that, in the present dataset, the predefined physiological category structure was not strongly supported as the latent basis of item responses. However, further refinement of low-discrimination items and future criterion-validity studies are needed before broader clinical implementation.

**7. Upon reviewing the data integrity, inconsistencies were noted between the statistics reported in Figure 1 and Table 2 for the same models. Specifically, the AIC for the unidimensional model varies from 29,180 to 29,077, while its log-likelihood ranges from -14,490 to -14,388; this suggests a potential discrepancy arising from different analysis stages or software executions. This inconsistency extends to the Results section, where the unidimensional model is described as having the best fit and the lowest log-likelihood. However, the data in Table 2 indicate that the two-factor Bifactor model is superior in terms of both AIC (28,966) and logLik (-14,283). Therefore, further clarification is required regarding the criteria used to prioritize BIC parsimony over a more complex model structure that appears to offer a better statistical fit.**

**Response:** We thank the reviewer for carefully identifying this point. We would like to clarify that the fit statistics presented in Figure 1 and Table 2 were derived from different analytical stages and are therefore not directly comparable. Specifically, Figure 1 summarizes the results of the exploratory factor analysis (EFA), whereas Table 2 reports the results of confirmatory factor analysis (CFA) within the MIRT framework. The differences in AIC and log-likelihood values reflect these distinct modeling approaches rather than inconsistencies in the data. To avoid confusion, we have revised the corresponding text in the Results section to explicitly state that Figure 1 represents exploratory analysis, while Table 2 reflects confirmatory model fitting.

We have also revised the wording in the Results and Discussion to avoid overstating that the unidimensional model had the “best fit.” Instead, we now clarify that both the unidimensional and two-factor solutions were statistically plausible, with the two-factor bifactor model showing somewhat better fit according to AIC and log-likelihood, whereas the unidimensional model was adopted as the initial operational model because of its greater parsimony and interpretability for CAT prototype development.

**8. Although the parameter calibration is described in detail, items Q33, Q43, and Q50 show low discriminatory power, which suggests the need to reassess their relevance within the item bank. In addition, to strengthen methodological transparency, it is recommended that the authors provide the numerical values of the Zh fit statistics in a formal table, rather than relying solely on qualitative descriptions or supplementary figures.**

**Response:** We thank the reviewer for this constructive comment. We agree that the low discrimination observed for items Q33, Q43, and Q50 warrants further review of their suitability within the item bank. In the revised manuscript, we have clarified that these items will be treated as candidates for revision in future refinement of the ECG item bank, rather than being assumed to function equivalently to the remaining items. At the same time, because this study focused on initial prototype development, we retained them in the current analyses and interpreted them cautiously.

We also agree that the numerical values of the Zh fit statistics should be presented more transparently. Accordingly, we have added a formal supplementary table reporting the item-level Zh statistics, rather than relying only on descriptive text and supplementary figures. We have revised the Methods/Results and Discussion sections to reflect these changes.

**Results:** Using Zh statistics, we evaluated item and person fit. Item-level Zh values are provided in Supplementary table S3 to improve transparency. Overall, the Zh statistics did not indicate marked item misfit, although several items with low discrimination were identified as candidates for future revision. Person-fit Zh statistics were centered around zero with a near-normal distribution, reflecting variable model fit across individuals (Supplemental figure S3A).

**Discussion:** Although overall item discrimination was strong, three items (Q33, Q43, and Q50) showed relatively low discrimination. These items should be considered candidates for revision or replacement during future expansion of the ECG item bank, because poorly discriminating items may reduce the precision of ability estimation in CAT-based assessment. In the present study, we retained these items because our goal was initial prototype development using the existing validated 50-item set; however, their performance will need to be reassessed in subsequent iterations.

**Supplemental table S3:** Item-level Zh fit statistics for the final unidimensional model

**9. Figure 3A shows two items whose behavior deviates drastically from the expected correlation (outliers) between difficulty and success rate. Given the clinical nature of the study, it is imperative to describe the content of these items. Identifying whether they involved tracings with artifacts or ambiguous diagnoses is crucial for refining the item bank.**

**Response:** We thank the reviewer for this valuable comment. We agree that the two outlier items in Figure 3A should be described more explicitly, because understanding whether these items reflect ambiguity, atypical clinical presentation, or other content-related features is important for refining the ECG item bank. In response, we identified the two outlier items in Figure 3A as [Q33] and [Q50] and added possible reasons for their deviation from the expected relationship between item difficulty and correct response rate. We also clarified in the Discussion that such outlier items require careful review during future refinement of the item bank, particularly if they involve atypical tracings, ambiguous diagnostic cues, or other features that may reduce their psychometric consistency.

**Results:** Figure 3 shows a significant negative correlation between item difficulty and correct response rate, confirming expected relationships. However, two items ([Q33] and [Q50]) deviated from this overall pattern.

**Discussion:** In addition, two of these items (Q33 and Q50) were also identified as outliers in the relationship between item difficulty and correct response rate (Figure 3A). Such deviations may reflect atypical ECG tracings, ambiguity in diagnostic interpretation, or item-specific features that are not fully captured by the overall difficulty parameter. These items therefore merit qualitative review in addition to psychometric evaluation and should be carefully examined during future refinement of the ECG item bank.

**10. The discussion emphasizes the usefulness of the unidimensional model, but it would benefit from a deeper analysis of why the models based on physiological categories showed poor fit. It would be valuable to consider whether this outcome is due to an uneven distribution of items across categories or whether ECG interpretation truly reflects an integrated, non-fragmented cognitive process. Furthermore, although items Q33, Q43, and Q50 were identified as having low discriminatory power, the discussion does not outline an action plan for them; it is important to note that in psychometrics, items with low discrimination can bias the estimation of ability, making it necessary to evaluate whether their inclusion in the item bank is appropriate. Finally, while the psychometric efficiency of the CAT prototype is evident, the manuscript should also address the need for future criterion validity studies that compare system results with the actual clinical performance of professionals in direct patient care settings.**

**Response:** We thank the reviewer for this thoughtful and constructive comment. We agree that the poor fit of the models based on physiological categories warrants more careful interpretation. In the revised manuscript, we have clarified that our findings do not exclude the possibility that ECG interpretation may have multidimensional aspects. Rather, the present results suggest that, in this dataset, the predefined physiological category structure was not strongly supported as the latent structure underlying item responses. This may reflect either the uneven distribution of items across categories or the possibility that ECG interpretation operates, at least in part, as a more integrated cognitive process in practical assessment settings. We have revised the Discussion accordingly to avoid overstatement.

We also agree that the low-discrimination items (Q33, Q43, and Q50) require an explicit action plan. In the revised manuscript, we now state that these items will be treated as candidates for review, revision, or replacement during future refinement of the ECG item bank, because poorly discriminating items may bias ability estimation in CAT-based assessment. However, because the present study focused on initial prototype development using the existing 50-item test set, these items were retained in the current calibration and interpreted cautiously.

Finally, we agree that future criterion validity studies are needed. We have therefore added a statement to the Discussion noting that subsequent work should compare CAT-based ability estimates with actual clinical performance in real-world patient-care settings in order to establish criterion-related validity.

**Discussion:** In this study, MIRT suggested that a unidimensional model was the most practical operational framework for the present item bank, although a two-factor solution was also statistically plausible. Importantly, the poorer fit of models based on predefined physiological categories does not necessarily mean that ECG interpretation lacks multidimensional aspects. Rather, our findings suggest that, in this dataset, the predefined physiological classification was not strongly supported as the latent structure underlying item responses. This may reflect the uneven distribution of items across categories, the overlap of cognitive processes required across ECG diagnoses, or the possibility that ECG interpretation functions as a more integrated, non-fragmented competency in practical assessment settings.

Although overall item discrimination was strong, three items (Q33, Q43, and Q50) showed relatively low discrimination. These items should be considered candidates for revision or replacement during future expansion of the ECG item bank, because poorly discriminating items may reduce the precision of ability estimation in CAT-based assessment. In the present study, we retained these items because our goal was initial prototype development using the existing validated 50-item set; however, their performance will need to be reassessed in subsequent iterations.

Although the present findings support the psychometric feasibility of the CAT prototype, additional studies are needed. In particular, future studies should examine criterion-related validity by comparing CAT-based ability estimates with actual clinical performance in real-world patient-care settings.

**Conclusion:** These findings support the feasibility of a CAT-based approach for assessing ECG interpretation skills and suggest that, in the present dataset, the predefined physiological category structure was not strongly supported as the latent basis of item responses. However, further refinement of low-discrimination items and future criterion-validity studies are needed before broader clinical implementation.

**11. The limitations are well identified, but they should also include the potential lack of international representativeness of the sample, given that recruitment was conducted primarily in Japan. The conclusion states that the CAT may enable standardized evaluation, but this should be qualified by noting that it remains a preliminary prototype not yet validated in clinical practice. Likewise, the assertion that the unidimensional model provides the “best fit” should be nuanced and presented as a “practical simplification for the initial prototype,” while acknowledging that the fit indices (AIC) suggest the multidimensional nature of ECG interpretation has not yet been fully captured by the current model.**

**Response:** We thank the reviewer for this insightful comment. We have revised the Limitations section to include the potential lack of international representativeness, as participants were primarily recruited in Japan.

We also clarified that the CAT system is a preliminary prototype that has not yet been validated in real-world or clinical settings.

In addition, we have revised the Conclusions to present the unidimensional model more cautiously as a practical simplification and to avoid overstatement of model fit.

**12. The example item in Supplemental Figure 1 illustrates a critical flaw in item construction. The clinical vignette presents a loss of consciousness and a heart rate of 22 bpm, findings that share a logical clinical correlation. However, a fundamental physiopathological dilemma arises extreme bradycardia in the context of hyperkalemia characteristically occurs when the condition is severe, a stage where the QRS complex is expected to be very wide and bizarre. In contrast, this tracing shows a QRS duration under 120 ms, lacking the markers of severity required to explain such profound clinical compromise solely through potassium toxicity.

Consequently, an experienced physician might observe the small deflection preceding the QRS in the frontal leads—suggesting a low-amplitude P-wave and a prolonged PR interval—and reasonably prioritize a diagnosis of extreme sinus bradycardia as the most consistent explanation for the clinical data, even if hyperkalemia is present. While some might still argue for hyperkalemia, this level of clinical debate is precisely what a standardized assessment should avoid. For a Computerized Adaptive Testing (CAT) system to be valid, the diagnostics must be unequivocal and resolved beforehand. Any item that allows for competing, clinically sound interpretations penalizes advanced reasoning and introduces noise that undermines the reliability of the ability estimation**

**Response:** We thank the reviewer for this insightful and important comment regarding the example item. We agree that ambiguity in ECG interpretation can compromise the validity of assessment items, particularly in the context of computerized adaptive testing (CAT), where unambiguous diagnostic targets are essential.

With regard to the original ECG, the tracing may be interpreted as early-stage hyperkalemia with marked bradycardia, possibly related to sinus arrest. The absence of clearly identifiable sinus P waves across leads is not fully consistent with sinus bradycardia. However, certain waveform features—such as small deflections in the limb leads—could allow for alternative clinically reasonable interpretations.

In response, we have replaced the example shown in Supplemental Figure 1 with a revised ECG item that demonstrates more clearly defined and unequivocal electrocardiographic findings. The updated example avoids potential competing interpretations, such as those noted by the reviewer, and ensures that the correct diagnosis is more directly supported by the ECG features.

Given this potential for interpretive variability, we agree that the original item may not be optimal for use in a standardized assessment setting such as CAT. In addition, we will further refine our item review process in future studies to minimize the inclusion of questions that may allow for multiple clinically reasonable interpretations, thereby improving the reliability of the assessment.

We believe that the revised example enhances the clarity of the item, and that these considerations will contribute to improving the overall quality of the assessment tool in future iterations.

**13. Finally, I suggest that the authors enrich the article by including the following bibliographic references:**

Carmona-Puerta R, Lorenzo-Martínez E. The issue of electrocardiography interpretation competence revisited. Educación Médica Superior. 2025; 39: e4563. Available from: <https://ems.sld.cu/index.php/ems/article/view/4563/1612>

Carmona-Puerta R, Lorenzo-Martínez E. Electrocardiography teaching methods. Educación Médica Superior. 2025; 39: e4558. Available from: <https://ems.sld.cu/index.php/ems/article/view/4558/1610>

**Response:** We thank the reviewer for this valuable suggestion. We have incorporated the recommended references into the Introduction to better contextualize the current challenges in ECG interpretation competency and education. These additions strengthen the background and highlight the relevance of our study.

Thank you again for the thorough review of our work. We hope this manuscript is now acceptable for publication in *PLOS One*.

Lastly, we would like to add Hiroshi Kawakami listed below as a co-author for his contributions to this study, including revision.

Hiroshi Kawakami, MD

Department of Cardiology, Pulmonology, Hypertension and Nephrology, Ehime University Graduate School of Medicine, Toon, Japan.

Sincerely,

Shinji Inaba, MD, PhD

Department of Cardiology, Pulmonology, Hypertension and Nephrology, Ehime University Graduate School of Medicine, Toon, Ehime 791-0295, Japan

Phone: +81 89-960-5303; Fax: +81 89-960-5306; E-mail: inaba226@gmail.com
