# Supplementary material for: Development and initial validation of a computerized adaptive test prototype for ECG interpretation using item response theory
Source: PLoS One. 2026 Jul 17;21(7):e0344341. doi: 10.1371/journal.pone.0344341 (PMC13379108; doi:10.1371/journal.pone.0344341)
Supplement: Attachment — Submitted filename: Respnse to Reviewers_PlosOne_0701.docx [file pone.0344341.s004.docx]

July 02, 2026

**Johanna Pruller, Ph.D.**

Senior Editor

PLOS One

RE: PONE-D-25-57504R1

**“Development and Initial Validation of a Computerized Adaptive Test Prototype for ECG Interpretation Using Item Response Theory”**

Dear Dr. Pruller,

Thank you for the review of our manuscript. We have responded to comments from the Reviewer in the revised manuscript as follows:

**Reviewer #1**

**The authors have successfully and thoroughly addressed all the comments and concerns raised during the first round of review. The manuscript has improved significantly, the methodology is sound, and I have no further comments. I am happy to recommend this manuscript for publication in PLOS One.**

**Response:** We sincerely thank the reviewer for the careful re-evaluation of our manuscript and for the positive and encouraging comments. We greatly appreciate your recognition that we have adequately addressed the concerns raised during the initial review and that the manuscript has been substantially improved. We are grateful for your recommendation for publication in *PLOS ONE*.

**Reviewer #2**

**interesting paper. Some issues should be added. briefly I think the paper is interesting but needs more data to fit clinicians**

**Response:** We sincerely thank the reviewer for the constructive comments and valuable suggestions. We have carefully revised the manuscript to improve its clarity, clinical relevance, and interpretability for physician readers.

**1. from the abstract to the paper the present work sounds really technical and hard to be understood from physicians. maybe it should be repharaed**

**Response:** We sincerely thank the reviewer for this valuable feedback. We agree that the original manuscript relied heavily on specialized jargon, making it difficult for practicing physicians to fully understand. In response to your suggestion, we thoroughly revised and simplified the technical descriptions throughout the manuscript, beginning with the Abstract. We also added explanatory context to improve accessibility for a clinical audience. These revisions have been incorporated throughout the Abstract, Introduction, Results, and Discussion sections.

**2. also the clinical aim of the introduction should be made more easy to be understood**

**Response:** Thank you for this specific and helpful suggestion. We agree that the clinical aim stated in the Introduction needed to be presented more clearly for medical readers.

Following your recommendation, we have revised the relevant section in the Introduction to simplify the explanation of our objectives, making it more accessible to a clinical audience.

**3. maybe a figure summarizing one process may be of great help**

**Response:** Thank you for this excellent and constructive suggestion. We agree that a visual summary of the process is highly beneficial for clinical readers. Following your recommendation, we have added a new figure (Figure 1) to the revised manuscript. This flowchart clearly summarizes the overall research process, spanning from the initial 50-item ECG examination and data collection from the 535 healthcare professionals, through the multidimensional IRT analysis and item calibration, to the final development of the Computerized Adaptive Test (CAT) prototype for efficient individualized competency assessment.

The overall study workflow is illustrated in Figure 1.

**Figure 1.** **Overview of the study workflow.**

Fifty ECG multiple-choice questions (MCQs) were developed through expert consensus using the RAND/UCLA Appropriateness Method. An online assessment was subsequently conducted among 535 healthcare professionals. The collected responses were analyzed using multidimensional item response theory (MIRT) to select the optimal model and calibrate item parameters, including difficulty and discrimination. These parameter estimates were then used to develop the ECG computerized adaptive test (ECG-CAT) prototype.

**4. the results are very hard to be understood from physicians and should been made more explainable**

**Response:** Thank you for this valuable comment. We revised the Results section to provide additional narrative explanations and clinical interpretations of the findings. In particular, we added explanatory text regarding the practical implications of the dimensionality analyses and model selection process so that readers can better understand the results.

**5. The 5-factor bifactor model reports a chi-square value of –69 with 0 degrees of freedom, which is not interpretable. This model should be explicitly labeled as non-identifiable, unstable, or unsuitable for inferential comparison. Presenting its chi-square statistic in the same way as the other models may mislead readers. I recommend either removing the chi-square result for this model or marking it as “not interpretable.”**

**Response:** We thank the reviewer for identifying this important issue. We have revised the Table 2 to clarify that the chi-square statistic for the five-factor bifactor model is **"not interpretable"** due to the model yielding zero degrees of freedom. This modification has been added to avoid potential misunderstanding by readers.

**6. The sample includes cardiologists, non-cardiologists, technologists, nurses, students, and other healthcare professionals. This heterogeneity is useful, but the manuscript only describes expertise categories descriptively. Given that item parameters can be influenced by subgroup composition, the authors should consider whether differential item functioning was assessed across relevant groups, particularly experts versus novices or physicians versus non-physicians. At minimum, the absence of DIF analysis should be acknowledged as a limitation.**

**Response:** We appreciate this insightful comment. We agree that differential item functioning (DIF) analysis would provide additional information regarding measurement invariance across professional groups. Because the primary objective of the present study was the initial calibration of the ECG item bank and development of a CAT prototype, DIF analysis was beyond the scope of the current investigation. However, we acknowledge this limitation and have added a statement to the Discussion section noting that future studies should evaluate DIF across relevant subgroups, including physicians versus non-physicians and experts versus novices.

**Limitations:** Fourth, differential item functioning (DIF) analysis was not performed to assess whether item parameters were invariant across participant subgroups. Consequently, potential measurement bias between subgroups cannot be excluded. Future studies should evaluate DIF to confirm measurement invariance and ensure the fairness of the ECG-CAT across diverse healthcare professionals.

Thank you again for the thorough review of our work. We hope this manuscript is now acceptable for publication in *PLOS One*.

Sincerely,

Shinji Inaba, MD, PhD

Department of Cardiology, Pulmonology, Hypertension and Nephrology, Ehime University Graduate School of Medicine, Toon, Ehime 791-0295, Japan

Phone: +81 89-960-5303; Fax: +81 89-960-5306; E-mail: inaba226@gmail.com
