# Supplementary material for: Development and initial validation of a computerized adaptive test prototype for ECG interpretation using item response theory
Source: PLoS One. 2026 Jul 17;21(7):e0344341. doi: 10.1371/journal.pone.0344341 (PMC13379108; doi:10.1371/journal.pone.0344341)
Supplement: Peer review history

# Peer review: Decision Letter 0

**DOI:** 10.1371/journal.pone.0344341.r001

**Authors:** Helen Howard

22 Mar 2026

PONE-D-25-57504

Development of a Computerized Adaptive Test for ECG Interpretation Using Item Response Theory

PLOS One

Dear Dr. Inaba,

Thank you for submitting your manuscript to PLOS ONE. After careful consideration, we feel that it has merit but does not fully meet PLOS ONE’s publication criteria as it currently stands. Therefore, we invite you to submit a revised version of the manuscript that addresses the points raised during the review process.

Please note that we have only been able to secure a single reviewer to assess your manuscript. We are issuing a decision on your manuscript at this point to prevent further delays in the evaluation of your manuscript. Please be aware that the editor who handles your revised manuscript might find it necessary to invite additional reviewers to assess this work once the revised manuscript is submitted. However, we will aim to proceed on the basis of this single review if possible.

Could you please revise the manuscript to carefully address the concerns raised?

Please submit your revised manuscript by May 06 2026 11:59PM. If you will need more time than this to complete your revisions, please reply to this message or contact the journal office at plosone@plos.org. When you're ready to submit your revision, log on to https://www.editorialmanager.com/pone/ and select the 'Submissions Needing Revision' folder to locate your manuscript file.

Please include the following items when submitting your revised manuscript:

- A letter that responds to each point raised by the academic editor and reviewer(s). You should upload this letter as a separate file labeled 'Response to Reviewers'.
- A marked-up copy of your manuscript that highlights changes made to the original version. You should upload this as a separate file labeled 'Revised Manuscript with Track Changes'.
- An unmarked version of your revised paper without tracked changes. You should upload this as a separate file labeled 'Manuscript'.

If you would like to make changes to your financial disclosure, please include your updated statement in your cover letter. Guidelines for resubmitting your figure files are available below the reviewer comments at the end of this letter.

If applicable, we recommend that you deposit your laboratory protocols in protocols.io to enhance the reproducibility of your results. Protocols.io assigns your protocol its own identifier (DOI) so that it can be cited independently in the future. For instructions see: https://journals.plos.org/plosone/s/submission-guidelines#loc-laboratory-protocols. Additionally, PLOS ONE offers an option for publishing peer-reviewed Lab Protocol articles, which describe protocols hosted on protocols.io. Read more information on sharing protocols at https://plos.org/protocols?utm_medium=editorial-email&utm_source=authorletters&utm_campaign=protocols.

We look forward to receiving your revised manuscript.

Kind regards,

Helen Howard

Staff Editor

PLOS One

Journal Requirements:

When submitting your revision, we need you to address these additional requirements.

1. Please ensure that your manuscript meets PLOS ONE's style requirements, including those for file naming. The PLOS ONE style templates can be found at

https://journals.plos.org/plosone/s/file?id=wjVg/PLOSOne_formatting_sample_main_body.pdf and

https://journals.plos.org/plosone/s/file?id=ba62/PLOSOne_formatting_sample_title_authors_affiliations.pdf

2. Please note that PLOS One has specific guidelines on code sharing for submissions in which author-generated code underpins the findings in the manuscript. In these cases, we expect all author-generated code to be made available without restrictions upon publication of the work. Please review our guidelines at https://journals.plos.org/plosone/s/materials-and-software-sharing#loc-sharing-code and ensure that your code is shared in a way that follows best practice and facilitates reproducibility and reuse.

3. Thank you for stating the following financial disclosure: [This study was funded by industrial seeds support from Ehime University, Ehime, Japan and by health care science institute, Tokyo, Japan.].

Please state what role the funders took in the study.  If the funders had no role, please state: ""The funders had no role in study design, data collection and analysis, decision to publish, or preparation of the manuscript.""

If this statement is not correct you must amend it as needed.

Please include this amended Role of Funder statement in your cover letter; we will change the online submission form on your behalf.

4. Please include captions for your Supporting Information files at the end of your manuscript, and update any in-text citations to match accordingly. Please see our Supporting Information guidelines for more information: http://journals.plos.org/plosone/s/supporting-information.

5. If the reviewer comments include a recommendation to cite specific previously published works, please review and evaluate these publications to determine whether they are relevant and should be cited. There is no requirement to cite these works unless the editor has indicated otherwise.

[Note: HTML markup is below. Please do not edit.]

Reviewers' comments:

Reviewer's Responses to Questions

**Comments to the Author**

1. Is the manuscript technically sound, and do the data support the conclusions?

The manuscript must describe a technically sound piece of scientific research with data that supports the conclusions. Experiments must have been conducted rigorously, with appropriate controls, replication, and sample sizes. The conclusions must be drawn appropriately based on the data presented.

Reviewer #1: Partly

**********

2. Has the statistical analysis been performed appropriately and rigorously?

Reviewer #1: No

**********

3. Have the authors made all data underlying the findings in their manuscript fully available?

The PLOS Data policy requires authors to make all data underlying the findings described in their manuscript fully available without restriction, with rare exception (please refer to the Data Availability Statement in the manuscript PDF file). The data should be provided as part of the manuscript or its supporting information, or deposited to a public repository. For example, in addition to summary statistics, the data points behind means, medians and variance measures should be available. If there are restrictions on publicly sharing data—e.g. participant privacy or use of data from a third party—those must be specified.

Reviewer #1: No

**********

4. Is the manuscript presented in an intelligible fashion and written in standard English?

PLOS ONE does not copyedit accepted manuscripts, so the language in submitted articles must be clear, correct, and unambiguous. Any typographical or grammatical errors should be corrected at revision, so please note any specific errors here.

Reviewer #1: Yes

**********

5. Review Comments to the Author

Please use the space provided to explain your answers to the questions above. You may also include additional comments for the author, including concerns about dual publication, research ethics, or publication ethics. (Please upload your review as an attachment if it exceeds 20,000 characters)

Reviewer #1: I have carefully read the full manuscript, and I must acknowledge that it is a study with genuine scientific merit and practical relevance in the field of medical education and electrocardiography. The application of MIRT and the development of a CAT prototype represent an innovative approach that can contribute meaningfully to the standardization of ECG competency assessment. However, there are several points to which the authors should pay attention:

1. The title adequately reflects the content of the study, but it could benefit from greater methodological precision. It is recommended to specify that this is an initial validation study or a development phase, to prevent readers from assuming that the CAT is already fully validated and operational.

2. The introduction provides a clear context for the clinical relevance of the study, but there is some redundancy in the explanation of MIRT and CAT. It is recommended to simplify and avoid repetition to improve the overall flow. The statement that “no MIRT tools for ECG exist” should be nuanced with broader references, since there are previous approaches in medical education which, although not identical, could be considered as antecedents.

3.While the Supplemental Methods provide a clearer description of the participant profiles and the recruitment process at Ehime University, the manuscript would still benefit from a detailed demographic table. Specifically, the exact proportion of experts versus novices should be reported, as a skewed distribution in clinical expertise can significantly influence the stability of the a and b parameters during MIRT calibration.

4. The decision to fix the guessing parameter (c) at 0.2 requires a stronger justification. In practice, the probability of guessing varies considerably depending on the ECG pattern—for example, identifying a Wolf-Parkinson-White tracing is not comparable to recognizing a simple sinus rhythm. Fixing cat a constant value may oversimplify the reality of visual interpretation. The authors should clarify why 0.2 was chosen, whether sensitivity analyses with alternative values were performed, and discuss whether an estimated parameter or a 2PL model might have provided a more realistic representation of the data.

5. A key assumption of IRT is that success on one item should not condition success on another. Since in an ECG one finding (e.g., peaked T waves) is often linked to another (e.g., QRS widening in hyperkalemia), the authors should confirm whether they performed local dependence tests (such as Yen’s Q3) to validate this assumption.

6. The reporting of model fit indices in Table 2 presents two critical inconsistencies. First, while the bifactor (2-factor) model shows superior fit according to AIC (28966 vs. 29077) and log-likelihood (–14283 vs. –14388), the unidimensional model was selected based primarily on BIC (29719 vs. 29823). From a methodological standpoint, AIC indicates that a specific factor structure may better explain the variance, and the authors should discuss why parsimony was prioritized over improved fit, especially given that ECG interpretation is clinically multidimensional. Second, the 5-factor bifactor model reports a chi-square value of –69 with 0 degrees of freedom, which is mathematically invalid and suggests either non-convergence of the estimation algorithm or an over-identified (saturated) model. This invalidates direct statistical comparison using chi-square tests. The authors should clarify whether the 5-factor model was discarded due to convergence issues or insufficient sample size (n = 535), and it is recommended to report this model as “non-convergent” or “misidentified” rather than presenting fit statistics that lack mathematical validity.

7. Upon reviewing the data integrity, inconsistencies were noted between the statistics reported in Figure 1 and Table 2 for the same models. Specifically, the AIC for the unidimensional model varies from 29,180 to 29,077, while its log-likelihood ranges from -14,490 to -14,388; this suggests a potential discrepancy arising from different analysis stages or software executions. This inconsistency extends to the Results section, where the unidimensional model is described as having the best fit and the lowest log-likelihood. However, the data in Table 2 indicate that the two-factor Bifactor model is superior in terms of both AIC (28,966) and logLik (-14,283). Therefore, further clarification is required regarding the criteria used to prioritize BIC parsimony over a more complex model structure that appears to offer a better statistical fit.

8. Although the parameter calibration is described in detail, items Q33, Q43, and Q50 show low discriminatory power, which suggests the need to reassess their relevance within the item bank. In addition, to strengthen methodological transparency, it is recommended that the authors provide the numerical values of the Zh fit statistics in a formal table, rather than relying solely on qualitative descriptions or supplementary figures.

9. Figure 3A shows two items whose behavior deviates drastically from the expected correlation (outliers) between difficulty and success rate. Given the clinical nature of the study, it is imperative to describe the content of these items. Identifying whether they involved tracings with artifacts or ambiguous diagnoses is crucial for refining the item bank.

10. The discussion emphasizes the usefulness of the unidimensional model, but it would benefit from a deeper analysis of why the models based on physiological categories showed poor fit. It would be valuable to consider whether this outcome is due to an uneven distribution of items across categories or whether ECG interpretation truly reflects an integrated, non-fragmented cognitive process. Furthermore, although items Q33, Q43, and Q50 were identified as having low discriminatory power, the discussion does not outline an action plan for them; it is important to note that in psychometrics, items with low discrimination can bias the estimation of ability, making it necessary to evaluate whether their inclusion in the item bank is appropriate. Finally, while the psychometric efficiency of the CAT prototype is evident, the manuscript should also address the need for future criterion validity studies that compare system results with the actual clinical performance of professionals in direct patient care settings.

11. The limitations are well identified, but they should also include the potential lack of international representativeness of the sample, given that recruitment was conducted primarily in Japan. The conclusion states that the CAT may enable standardized evaluation, but this should be qualified by noting that it remains a preliminary prototype not yet validated in clinical practice. Likewise, the assertion that the unidimensional model provides the “best fit” should be nuanced and presented as a “practical simplification for the initial prototype,” while acknowledging that the fit indices (AIC) suggest the multidimensional nature of ECG interpretation has not yet been fully captured by the current model.

12. The example item in Supplemental Figure 1 illustrates a critical flaw in item construction. The clinical vignette presents a loss of consciousness and a heart rate of 22 bpm, findings that share a logical clinical correlation. However, a fundamental physiopathological dilemma arises extreme bradycardia in the context of hyperkalemia characteristically occurs when the condition is severe, a stage where the QRS complex is expected to be very wide and bizarre. In contrast, this tracing shows a QRS duration under 120 ms, lacking the markers of severity required to explain such profound clinical compromise solely through potassium toxicity.

Consequently, an experienced physician might observe the small deflection preceding the QRS in the frontal leads—suggesting a low-amplitude P-wave and a prolonged PR interval—and reasonably prioritize a diagnosis of extreme sinus bradycardia as the most consistent explanation for the clinical data, even if hyperkalemia is present. While some might still argue for hyperkalemia, this level of clinical debate is precisely what a standardized assessment should avoid. For a Computerized Adaptive Testing (CAT) system to be valid, the diagnostics must be unequivocal and resolved beforehand. Any item that allows for competing, clinically sound interpretations penalizes advanced reasoning and introduces noise that undermines the reliability of the ability estimation

13. Finally, I suggest that the authors enrich the article by including the following bibliographic references:

Carmona-Puerta R, Lorenzo-Martínez E. The issue of electrocardiography interpretation competence revisited. Educación Médica Superior. 2025; 39: e4563. Available from: https://ems.sld.cu/index.php/ems/article/view/4563/1612

Carmona-Puerta R, Lorenzo-Martínez E. Electrocardiography teaching methods. Educación Médica Superior. 2025; 39: e4558. Available from: https://ems.sld.cu/index.php/ems/article/view/4558/1610

**********

6. PLOS authors have the option to publish the peer review history of their article (what does this mean?). If published, this will include your full peer review and any attached files.

If you choose “no”, your identity will remain anonymous but your review may still be made public.

**Do you want your identity to be public for this peer review?**  For information about this choice, including consent withdrawal, please see our Privacy Policy.

Reviewer #1: No

**********

[NOTE: If reviewer comments were submitted as an attachment file, they will be attached to this email and accessible via the submission site. Please log into your account, locate the manuscript record, and check for the action link "View Attachments". If this link does not appear, there are no attachment files.]

To ensure your figures meet our technical requirements, please review our figure guidelines: https://journals.plos.org/plosone/s/figures

You may also use PLOS’s free figure tool, NAAS, to help you prepare publication quality figures: https://journals.plos.org/plosone/s/figures#loc-tools-for-figure-preparation.

NAAS will assess whether your figures meet our technical requirements by comparing each figure against our figure specifications.

# Author comment: Author response to Decision Letter 1

**DOI:** 10.1371/journal.pone.0344341.r002

24 Apr 2026

April 23, 2026

Helen Howard, MD

Staff Editor

PLOS One

RE: PONE-D-25-57504

“Development of a Computerized Adaptive Test for ECG Interpretation Using Item Response Theory”

Dear Dr. Howard,

Thank you for the review of our manuscript. We have responded to comments from the Reviewer in the revised manuscript as follows:

Reviewer #1

I have carefully read the full manuscript, and I must acknowledge that it is a study with genuine scientific merit and practical relevance in the field of medical education and electrocardiography. The application of MIRT and the development of a CAT prototype represent an innovative approach that can contribute meaningfully to the standardization of ECG competency assessment. However, there are several points to which the authors should pay attention:

Response: We sincerely thank Reviewer #1 for the careful evaluation of our manuscript and for the insightful and constructive comments. We have revised the manuscript accordingly.

1. The title adequately reflects the content of the study, but it could benefit from greater methodological precision. It is recommended to specify that this is an initial validation study or a development phase, to prevent readers from assuming that the CAT is already fully validated and operational.

Response: We thank the reviewer for this important suggestion. We agree that the title should more clearly reflect the developmental nature of this study to avoid any misunderstanding that the CAT system is fully validated.

In response, we have revised the title to better emphasize that this study represents an initial development and validation phase. The revised title is as follows:

“Development and Initial Validation of a Computerized Adaptive Test Prototype for ECG Interpretation Using Item Response Theory”

2. The introduction provides a clear context for the clinical relevance of the study, but there is some redundancy in the explanation of MIRT and CAT. It is recommended to simplify and avoid repetition to improve the overall flow. The statement that “no MIRT tools for ECG exist” should be nuanced with broader references, since there are previous approaches in medical education which, although not identical, could be considered as antecedents.

Response: We thank the reviewer for this helpful comment. We have revised the Introduction to reduce redundancy, particularly in the descriptions of MIRT and CAT, and to improve overall clarity. We also incorporated the suggested references to better contextualize the current challenges in ECG education and assessment.

3. While the Supplemental Methods provide a clearer description of the participant profiles and the recruitment process at Ehime University, the manuscript would still benefit from a detailed demographic table. Specifically, the exact proportion of experts versus novices should be reported, as a skewed distribution in clinical expertise can significantly influence the stability of the a and b parameters during MIRT calibration.

Response: We thank the reviewer for this important suggestion. We have revised the manuscript to include a detailed description of participant demographics in the text, including their professional backgrounds.

In addition, we categorized participants according to their level of ECG expertise (e.g., experts, intermediate, and novices) and clarified their proportions in the revised manuscript.

We also acknowledge that the distribution of clinical expertise may influence the stability of item parameter estimates in MIRT and have added this point to the Discussion as a limitation.

Supplemental materials: A total of 535 participants were included in the analysis, comprising cardiologists (n=41), medical technologists (n=111), non-cardiologists (n=42), senior residents (n=13), junior residents (n=21), clinical engineers (n=31), medical students (n=77), paramedics (n=33), nurses (n=144), nursing students (n=8), and others (n=14). For descriptive purposes, participants were broadly categorized into three groups based on presumed ECG interpretation expertise: experts (e.g., cardiologists), intermediate (e.g., non-cardiologists, residents, and allied health professionals), and novices (e.g., students), (Supplementary table S1). The distribution of expertise levels was considered in the interpretation of MIRT parameter estimates.

Supplementary table S1. Participant Expertise Levels

Expertise level Occupation N=535 %

Expert Cardiologists 41 7.7

Intermediate Non-cardiologists, residents, nurses, technologists, engineers, paramedics 395 73.8

Novice Medical students, nursing students 85 15.9

Others/Unclassified Others 14 2.6

Limitation

The heterogeneous distribution of participant expertise may have influenced item parameter estimation, which should be considered when interpreting the results.

4. The decision to fix the guessing parameter (c) at 0.2 requires a stronger justification. In practice, the probability of guessing varies considerably depending on the ECG pattern—for example, identifying a Wolf-Parkinson-White tracing is not comparable to recognizing a simple sinus rhythm. Fixing cat a constant value may oversimplify the reality of visual interpretation. The authors should clarify why 0.2 was chosen, whether sensitivity analyses with alternative values were performed, and discuss whether an estimated parameter or a 2PL model might have provided a more realistic representation of the data.

Response: We thank the reviewer for this important comment. We agree that the probability of guessing is unlikely to be identical across ECG items. Upon revisiting our analysis, we realized that the manuscript wording was imprecise. In the mirt analysis, the value of 0.20 was used as an initial value based on the five-option multiple-choice format, but it was not fixed during estimation. The guessing parameter was estimated for each item, which is why the g values vary across items in Supplementary Table 2. We have revised the Methods and related text to clarify this point and to avoid the incorrect impression that the guessing parameter was fixed at 0.20 for all items.

Methods: Based on the exploratory factor analysis results, we estimated both unidimensional and bifactor models using confirmatory factor analysis within a three-parameter logistic (3PL) MIRT framework, fixing the guessing parameter at 0.2 to suit the binary response format. Because all items were five-option multiple-choice questions, the guessing parameter was initialized at 0.20 in the mirt model. This value was used only as a starting value for estimation and was not fixed. Accordingly, item-specific guessing parameters were estimated and allowed to vary across items.

Discussion: The probability of guessing differed across items, as expected, and was estimated rather than fixed in the final model. This approach better reflects the variability in item characteristics compared to assuming a constant guessing parameter.

5. A key assumption of IRT is that success on one item should not condition success on another. Since in an ECG one finding (e.g., peaked T waves) is often linked to another (e.g., QRS widening in hyperkalemia), the authors should confirm whether they performed local dependence tests (such as Yen’s Q3) to validate this assumption.

Response: We thank the reviewer for this important comment. We agree that local independence is a key assumption in IRT and that some ECG items may involve clinically related findings. In our analysis, local dependence among items was examined, with Cramer’s V < 0.2 indicating acceptable levels of dependence. We have clarified this point in the revised Methods section to make the assessment of local dependence more explicit.

Methods: Criterion (AIC), Bayesian Information Criterion (BIC), and root mean square error of approximation (RMSEA), with RMSEA < 0.08 considered acceptable.16-18 Local dependence among items was examined as part of the IRT assumption checks, with Cramer’s V < 0.2 indicating acceptable levels of dependence.19

Results: No problematic local dependence was identified according to the predefined criterion of Cramer’s V < 0.2.

6. The reporting of model fit indices in Table 2 presents two critical inconsistencies. First, while the bifactor (2-factor) model shows superior fit according to AIC (28966 vs. 29077) and log-likelihood (–14283 vs. –14388), the unidimensional model was selected based primarily on BIC (29719 vs. 29823). From a methodological standpoint, AIC indicates that a specific factor structure may better explain the variance, and the authors should discuss why parsimony was prioritized over improved fit, especially given that ECG interpretation is clinically multidimensional. Second, the 5-factor bifactor model reports a chi-square value of –69 with 0 degrees of freedom, which is mathematically invalid and suggests either non-convergence of the estimation algorithm or an over-identified (saturated) model. This invalidates direct statistical comparison using chi-square tests. The authors should clarify whether the 5-factor model was discarded due to convergence issues or insufficient sample size (n = 535), and it is recommended to report this model as “non-convergent” or “misidentified” rather than presenting fit statistics that lack mathematical validity.

Response: We thank the reviewer for this important comment. We agree that the two-factor model showed somewhat better fit according to AIC and log-likelihood, whereas the unidimensional model was favored by BIC. We have therefore revised the manuscript to clarify that the selection of the unidimensional model was not based on a single fit index alone. Rather, both unidimensional and two-factor structures were considered plausible from a statistical standpoint. However, after descriptively examining the two-factor solution, we found that it did not yield a clinically meaningful or sufficiently interpretable separation of latent domains for ECG competency assessment. Because the primary aim of this study was to develop an initial and practically implementable CAT prototype, we prioritized parsimony and interpretability and therefore adopted the unidimensional model as the initial operational model. We have revised the Abstract, Results, Discussion, and Conclusion accordingly.

Regarding the five-factor bifactor model, we agree that the reported chi-square value with 0 degrees of freedom is not interpretable for formal model comparison. We have therefore revised Table 2 and the related text to clarify that this model was not suitable for inferential chi-square comparison and should be interpreted cautiously as an unstable/misidentified solution rather than as evidence supporting or refuting that factor structure.

Abstract: A unidimensional model and a two-factor model both emerged as plausible latent structures.

Although MIRT analysis suggested that both unidimensional and two-factor models were plausible, and the unidimensional model was adopted as a practical and interpretable solution for the initial CAT prototype.

Results: Based on these results, we selected the unidimensional and two-factor models as candidates for further analysis. Model-comparison indices suggested that both unidimensional and bifactor two-factor solutions were plausible. The bifactor two-factor model showed somewhat better AIC and log-likelihood values, whereas the unidimensional model was favored by BIC. Because the primary purpose of the present study was to establish an initial, operationally simple framework for CAT prototype development, the unidimensional model was adopted for subsequent item calibration and CAT implementation.

The chi-square statistic for the bifactor five-factor model was not interpretable for formal statistical comparison because the model yielded 0 degrees of freedom, suggesting an unstable or misidentified solution in the present sample. Accordingly, this model was not used as a basis for inferential model selection.

Discussion: An evaluation using MIRT on the ECG online test results revealed that both unidimensional and two-factor models emerged as statistically plausible latent structures. However, although the two-factor model showed slightly improved fit on some statistical indices, descriptive examination did not support a clinically meaningful or practically useful separation of ECG competency domains. Therefore, for the purpose of initial CAT prototype development, we adopted the unidimensional model as a more parsimonious and interpretable operational framework. These findings suggest that, at least for the present item bank, ECG interpretation competency may be operationalized as a largely integrated latent trait for practical assessment purposes.

Conclusions: MIRT analysis suggested that a unidimensional model provided the plausible fit among the candidate latent factors, although this may represent a practical simplification for the initial prototype. Furthermore, the analysis enabled the precise calibration of difficulty levels for the ECG MCQs. Using the parameter estimates obtained through MIRT analysis, we successfully developed a preliminary prototype CAT system for assessing ECG interpretation skills. These findings support the feasibility of a CAT-based approach for assessing ECG interpretation skills and suggest that, in the present dataset, the predefined physiological category structure was not strongly supported as the latent basis of item responses. However, further refinement of low-discrimination items and future criterion-validity studies are needed before broader clinical implementation.

7. Upon reviewing the data integrity, inconsistencies were noted between the statistics reported in Figure 1 and Table 2 for the same models. Specifically, the AIC for the unidimensional model varies from 29,180 to 29,077, while its log-likelihood ranges from -14,490 to -14,388; this suggests a potential discrepancy arising from different analysis stages or software executions. This inconsistency extends to the Results section, where the unidimensional model is described as having the best fit and the lowest log-likelihood. However, the data in Table 2 indicate that the two-factor Bifactor model is superior in terms of both AIC (28,966) and logLik (-14,283). Therefore, further clarification is required regarding the criteria used to prioritize BIC parsimony over a more complex model structure that appears to offer a better statistical fit.

Response: We thank the reviewer for carefully identifying this point. We would like to clarify that the fit statistics presented in Figure 1 and Table 2 were derived from different analytical stages and are therefore not directly comparable. Specifically, Figure 1 summarizes the results of the exploratory factor analysis (EFA), whereas Table 2 reports the results of confirmatory factor analysis (CFA) within the MIRT framework. The differences in AIC and log-likelihood values reflect these distinct modeling approaches rather than inconsistencies in the data. To avoid confusion, we have revised the corresponding text in the Results section to explicitly state that Figure 1 represents exploratory analysis, while Table 2 reflects confirmatory model fitting.

We have also revised the wording in the Results and Discussion to avoid overstating that the unidimensional model had the “best fit.” Instead, we now clarify that both the unidimensional and two-factor solutions were statistically plausible, with the two-factor bifactor model showing somewhat better fit according to AIC and log-likelihood, whereas the unidimensional model was adopted as the initial operational model because of its greater parsimony and interpretability for CAT prototype development.

8. Although the parameter calibration is described in detail, items Q33, Q43, and Q50 show low discriminatory power, which suggests the need to reassess their relevance within the item bank. In addition, to strengthen methodological transparency, it is recommended that the authors provide the numerical values of the Zh fit statistics in a formal table, rather than relying solely on qualitative descriptions or supplementary figures.

Response: We thank the reviewer for this constructive comment. We agree that the low discrimination observed for items Q3

Attachment Submitted filename: Respnse to Reviewers_PlosOne_0423.docx

# Peer review: Decision Letter 1

**DOI:** 10.1371/journal.pone.0344341.r003

**Authors:** Johanna Pruller

22 Jun 2026

<div>PONE-D-25-57504R1

Development and Initial Validation of a Computerized Adaptive Test Prototype for ECG Interpretation Using Item Response Theory

PLOS One

Dear Dr. Inaba,

Thank you for submitting your manuscript to PLOS ONE. After careful consideration, we feel that it has merit but does not fully meet PLOS ONE’s publication criteria as it currently stands. Therefore, we invite you to submit a revised version of the manuscript that addresses the points raised during the review process.

The manuscript has been evaluated by two reviewers, and their comments are available below.

The reviewers have raised a number of concerns that need attention. Could you please revise the manuscript to carefully address the concerns raised?

Please submit your revised manuscript by Aug 06 2026 11:59PM. If you will need more time than this to complete your revisions, please reply to this message or contact the journal office at plosone@plos.org. When you're ready to submit your revision, log on to https://www.editorialmanager.com/pone/ and select the 'Submissions Needing Revision' folder to locate your manuscript file.

Please include the following items when submitting your revised manuscript:

- A letter that responds to each point raised by the academic editor and reviewer(s). You should upload this letter as a separate file labeled 'Response to Reviewers'.
- A marked-up copy of your manuscript that highlights changes made to the original version. You should upload this as a separate file labeled 'Revised Manuscript with Track Changes'.
- An unmarked version of your revised paper without tracked changes. You should upload this as a separate file labeled 'Manuscript'.

If you would like to make changes to your financial disclosure, please include your updated statement in your cover letter. Guidelines for resubmitting your figure files are available below the reviewer comments at the end of this letter.

If applicable, we recommend that you deposit your laboratory protocols in protocols.io to enhance the reproducibility of your results. Protocols.io assigns your protocol its own identifier (DOI) so that it can be cited independently in the future. For instructions see: https://journals.plos.org/plosone/s/submission-guidelines#loc-laboratory-protocols. Additionally, PLOS ONE offers an option for publishing peer-reviewed Lab Protocol articles, which describe protocols hosted on protocols.io. Read more information on sharing protocols at https://plos.org/protocols?utm_medium=editorial-email&utm_source=authorletters&utm_campaign=protocols.

As the corresponding author, your ORCID iD is verified in the submission system and will appear in the published article. PLOS supports the use of ORCID, and we encourage all coauthors to register for an ORCID iD and use it as well. Please encourage your coauthors to verify their ORCID iD within the submission system before final acceptance, as unverified ORCID iDs will not appear in the published article. *Only*  the individual author can complete the verification step; PLOS staff *cannot*  verify ORCID iDs on behalf of authors.

We look forward to receiving your revised manuscript.

Kind regards,

Johanna Pruller, Ph.D.

Senior Editor

PLOS One

Journal Requirements:

If the reviewer comments include a recommendation to cite specific previously published works, please review and evaluate these publications to determine whether they are relevant and should be cited. There is no requirement to cite these works unless the editor has indicated otherwise.

[Note: HTML markup is below. Please do not edit.]

Reviewers' comments:

Reviewer's Responses to Questions

**Comments to the Author**

1. If the authors have adequately addressed your comments raised in a previous round of review and you feel that this manuscript is now acceptable for publication, you may indicate that here to bypass the “Comments to the Author” section, enter your conflict of interest statement in the “Confidential to Editor” section, and submit your "Accept" recommendation.

Reviewer #1: All comments have been addressed

Reviewer #2: (No Response)

**********

2. Is the manuscript technically sound, and do the data support the conclusions?

The manuscript must describe a technically sound piece of scientific research with data that supports the conclusions. Experiments must have been conducted rigorously, with appropriate controls, replication, and sample sizes. The conclusions must be drawn appropriately based on the data presented.

Reviewer #1: Yes

Reviewer #2: Yes

**********

3. Has the statistical analysis been performed appropriately and rigorously?

Reviewer #1: Yes

Reviewer #2: Yes

**********

4. Have the authors made all data underlying the findings in their manuscript fully available?

The PLOS Data policy requires authors to make all data underlying the findings described in their manuscript fully available without restriction, with rare exception (please refer to the Data Availability Statement in the manuscript PDF file). The data should be provided as part of the manuscript or its supporting information, or deposited to a public repository. For example, in addition to summary statistics, the data points behind means, medians and variance measures should be available. If there are restrictions on publicly sharing data—e.g. participant privacy or use of data from a third party—those must be specified.

Reviewer #1: Yes

Reviewer #2: Yes

**********

5. Is the manuscript presented in an intelligible fashion and written in standard English?

PLOS ONE does not copyedit accepted manuscripts, so the language in submitted articles must be clear, correct, and unambiguous. Any typographical or grammatical errors should be corrected at revision, so please note any specific errors here.

Reviewer #1: Yes

Reviewer #2: Yes

**********

6. Review Comments to the Author

Please use the space provided to explain your answers to the questions above. You may also include additional comments for the author, including concerns about dual publication, research ethics, or publication ethics. (Please upload your review as an attachment if it exceeds 20,000 characters)

Reviewer #1: The authors have successfully and thoroughly addressed all the comments and concerns raised during the first round of review. The manuscript has improved significantly, the methodology is sound, and I have no further comments. I am happy to recommend this manuscript for publication in PLOS One.

Reviewer #2: interesting paper. Some issues should be added. briefly I think the paper is interesting but needs more data to fit clinicians

1) from the abstract to the paper the present work sounds really technical and hard to be understood from physicians. maybe it should be repharaed

2) also the clinical aim of the introduction should be made more easy to be understood

3)maybe a figure summarizing one process may be of great help

4) the results are very hard to be understood from physicians and should been made more expkainable

5) The 5-factor bifactor model reports a chi-square value of –69 with 0 degrees of freedom, which is not interpretable. This model should be explicitly labeled as non-identifiable, unstable, or unsuitable for inferential comparison. Presenting its chi-square statistic in the same way as the other models may mislead readers. I recommend either removing the chi-square result for this model or marking it as “not interpretable.”

6) The sample includes cardiologists, non-cardiologists, technologists, nurses, students, and other healthcare professionals. This heterogeneity is useful, but the manuscript only describes expertise categories descriptively. Given that item parameters can be influenced by subgroup composition, the authors should consider whether differential item functioning was assessed across relevant groups, particularly experts versus novices or physicians versus non-physicians. At minimum, the absence of DIF analysis should be acknowledged as a limitation.

**********

7. PLOS authors have the option to publish the peer review history of their article (what does this mean?). If published, this will include your full peer review and any attached files.

If you choose “no”, your identity will remain anonymous but your review may still be made public.

**Do you want your identity to be public for this peer review?**  For information about this choice, including consent withdrawal, please see our Privacy Policy.

Reviewer #1: No

Reviewer #2: **Yes:**  Fabrizio D'Ascenzo

**********

[NOTE: If reviewer comments were submitted as an attachment file, they will be attached to this email and accessible via the submission site. Please log into your account, locate the manuscript record, and check for the action link "View Attachments". If this link does not appear, there are no attachment files.]

To ensure your figures meet our technical requirements, please review our figure guidelines: https://journals.plos.org/plosone/s/figures

You may also use PLOS’s free figure tool, NAAS, to help you prepare publication quality figures: https://journals.plos.org/plosone/s/figures#loc-tools-for-figure-preparation.

NAAS will assess whether your figures meet our technical requirements by comparing each figure against our figure specifications.

# Author comment: Author response to Decision Letter 2

**DOI:** 10.1371/journal.pone.0344341.r004

1 Jul 2026

July 02, 2026

Johanna Pruller, Ph.D.

Senior Editor

PLOS One

RE: PONE-D-25-57504R1

“Development and Initial Validation of a Computerized Adaptive Test Prototype for ECG Interpretation Using Item Response Theory”

Dear Dr. Pruller,

Thank you for the review of our manuscript. We have responded to comments from the Reviewer in the revised manuscript as follows:

Reviewer #1

The authors have successfully and thoroughly addressed all the comments and concerns raised during the first round of review. The manuscript has improved significantly, the methodology is sound, and I have no further comments. I am happy to recommend this manuscript for publication in PLOS One.

Response: We sincerely thank the reviewer for the careful re-evaluation of our manuscript and for the positive and encouraging comments. We greatly appreciate your recognition that we have adequately addressed the concerns raised during the initial review and that the manuscript has been substantially improved. We are grateful for your recommendation for publication in PLOS ONE.

Reviewer #2

interesting paper. Some issues should be added. briefly I think the paper is interesting but needs more data to fit clinicians

Response: We sincerely thank the reviewer for the constructive comments and valuable suggestions. We have carefully revised the manuscript to improve its clarity, clinical relevance, and interpretability for physician readers.

1. from the abstract to the paper the present work sounds really technical and hard to be understood from physicians. maybe it should be repharaed

Response: We sincerely thank the reviewer for this valuable feedback. We agree that the original manuscript relied heavily on specialized jargon, making it difficult for practicing physicians to fully understand. In response to your suggestion, we thoroughly revised and simplified the technical descriptions throughout the manuscript, beginning with the Abstract. We also added explanatory context to improve accessibility for a clinical audience. These revisions have been incorporated throughout the Abstract, Introduction, Results, and Discussion sections.

2. also the clinical aim of the introduction should be made more easy to be understood

Response: Thank you for this specific and helpful suggestion. We agree that the clinical aim stated in the Introduction needed to be presented more clearly for medical readers.

Following your recommendation, we have revised the relevant section in the Introduction to simplify the explanation of our objectives, making it more accessible to a clinical audience.

3. maybe a figure summarizing one process may be of great help

Response: Thank you for this excellent and constructive suggestion. We agree that a visual summary of the process is highly beneficial for clinical readers. Following your recommendation, we have added a new figure (Figure 1) to the revised manuscript. This flowchart clearly summarizes the overall research process, spanning from the initial 50-item ECG examination and data collection from the 535 healthcare professionals, through the multidimensional IRT analysis and item calibration, to the final development of the Computerized Adaptive Test (CAT) prototype for efficient individualized competency assessment.

The overall study workflow is illustrated in Figure 1.

Figure 1. Overview of the study workflow.

Fifty ECG multiple-choice questions (MCQs) were developed through expert consensus using the RAND/UCLA Appropriateness Method. An online assessment was subsequently conducted among 535 healthcare professionals. The collected responses were analyzed using multidimensional item response theory (MIRT) to select the optimal model and calibrate item parameters, including difficulty and discrimination. These parameter estimates were then used to develop the ECG computerized adaptive test (ECG-CAT) prototype.

4. the results are very hard to be understood from physicians and should been made more explainable

Response: Thank you for this valuable comment. We revised the Results section to provide additional narrative explanations and clinical interpretations of the findings. In particular, we added explanatory text regarding the practical implications of the dimensionality analyses and model selection process so that readers can better understand the results.

5. The 5-factor bifactor model reports a chi-square value of –69 with 0 degrees of freedom, which is not interpretable. This model should be explicitly labeled as non-identifiable, unstable, or unsuitable for inferential comparison. Presenting its chi-square statistic in the same way as the other models may mislead readers. I recommend either removing the chi-square result for this model or marking it as “not interpretable.”

Response: We thank the reviewer for identifying this important issue. We have revised the Table 2 to clarify that the chi-square statistic for the five-factor bifactor model is "not interpretable" due to the model yielding zero degrees of freedom. This modification has been added to avoid potential misunderstanding by readers.

6. The sample includes cardiologists, non-cardiologists, technologists, nurses, students, and other healthcare professionals. This heterogeneity is useful, but the manuscript only describes expertise categories descriptively. Given that item parameters can be influenced by subgroup composition, the authors should consider whether differential item functioning was assessed across relevant groups, particularly experts versus novices or physicians versus non-physicians. At minimum, the absence of DIF analysis should be acknowledged as a limitation.

Response: We appreciate this insightful comment. We agree that differential item functioning (DIF) analysis would provide additional information regarding measurement invariance across professional groups. Because the primary objective of the present study was the initial calibration of the ECG item bank and development of a CAT prototype, DIF analysis was beyond the scope of the current investigation. However, we acknowledge this limitation and have added a statement to the Discussion section noting that future studies should evaluate DIF across relevant subgroups, including physicians versus non-physicians and experts versus novices.

Limitations: Fourth, differential item functioning (DIF) analysis was not performed to assess whether item parameters were invariant across participant subgroups. Consequently, potential measurement bias between subgroups cannot be excluded. Future studies should evaluate DIF to confirm measurement invariance and ensure the fairness of the ECG-CAT across diverse healthcare professionals.

Thank you again for the thorough review of our work. We hope this manuscript is now acceptable for publication in PLOS One.

Sincerely,

Shinji Inaba, MD, PhD

Department of Cardiology, Pulmonology, Hypertension and Nephrology, Ehime University Graduate School of Medicine, Toon, Ehime 791-0295, Japan

Phone: +81 89-960-5303; Fax: +81 89-960-5306; E-mail: inaba226@gmail.com

Attachment Submitted filename: Respnse to Reviewers_PlosOne_0701.docx

# Peer review: Decision Letter 2

**DOI:** 10.1371/journal.pone.0344341.r005

**Authors:** Leander Klein

6 Jul 2026

Development and Initial Validation of a Computerized Adaptive Test Prototype for ECG Interpretation Using Item Response Theory

PONE-D-25-57504R2

Dear Dr. Shinji Inaba,

We’re pleased to inform you that your manuscript has been judged scientifically suitable for publication and will be formally accepted for publication once it meets all outstanding technical requirements.

Within one week, you’ll receive an e-mail detailing the required amendments. When these have been addressed, you’ll receive a formal acceptance letter and your manuscript will be scheduled for publication.

An invoice will be generated when your article is formally accepted. Please note, if your institution has a publishing partnership with PLOS and your article meets the relevant criteria, all or part of your publication costs will be covered. Please make sure your user information is up-to-date by logging into Editorial Manager at Editorial Manager® and clicking the ‘Update My Information' link at the top of the page. For questions related to billing, please contact billing support.

If your institution or institutions have a press office, please notify them about your upcoming paper to help maximize its impact. If they’ll be preparing press materials, please inform our press team as soon as possible -- no later than 48 hours after receiving the formal acceptance. Your manuscript will remain under strict press embargo until 2 pm Eastern Time on the date of publication. For more information, please contact onepress@plos.org.

Kind regards,

Leander Luiz Klein, Ph.D.

Academic Editor

PLOS One

Additional Editor Comments (optional):

The current version of the manuscript was improved according the suggestions of the reviewers. Both of them suggested to accept the article with no additional sugestions.

**Comments to the Author**

1. If the authors have adequately addressed your comments raised in a previous round of review and you feel that this manuscript is now acceptable for publication, you may indicate that here to bypass the “Comments to the Author” section, enter your conflict of interest statement in the “Confidential to Editor” section, and submit your "Accept" recommendation.

Reviewer #1: All comments have been addressed

Reviewer #2: All comments have been addressed

**********

2. Is the manuscript technically sound, and do the data support the conclusions?

The manuscript must describe a technically sound piece of scientific research with data that supports the conclusions. Experiments must have been conducted rigorously, with appropriate controls, replication, and sample sizes. The conclusions must be drawn appropriately based on the data presented.

Reviewer #1: Yes

Reviewer #2: (No Response)

**********

3. Has the statistical analysis been performed appropriately and rigorously?

Reviewer #1: Yes

Reviewer #2: (No Response)

**********

4. Have the authors made all data underlying the findings in their manuscript fully available?

The PLOS Data policy requires authors to make all data underlying the findings described in their manuscript fully available without restriction, with rare exception (please refer to the Data Availability Statement in the manuscript PDF file). The data should be provided as part of the manuscript or its supporting information, or deposited to a public repository. For example, in addition to summary statistics, the data points behind means, medians and variance measures should be available. If there are restrictions on publicly sharing data—e.g. participant privacy or use of data from a third party—those must be specified.

Reviewer #1: Yes

Reviewer #2: (No Response)

**********

5. Is the manuscript presented in an intelligible fashion and written in standard English?

PLOS ONE does not copyedit accepted manuscripts, so the language in submitted articles must be clear, correct, and unambiguous. Any typographical or grammatical errors should be corrected at revision, so please note any specific errors here.

Reviewer #1: Yes

Reviewer #2: (No Response)

**********

6. Review Comments to the Author

Please use the space provided to explain your answers to the questions above. You may also include additional comments for the author, including concerns about dual publication, research ethics, or publication ethics. (Please upload your review as an attachment if it exceeds 20,000 characters)

Reviewer #1: (No Response)

Reviewer #2: (No Response)

**********

7. PLOS authors have the option to publish the peer review history of their article (what does this mean?). If published, this will include your full peer review and any attached files.

If you choose “no”, your identity will remain anonymous but your review may still be made public.

**Do you want your identity to be public for this peer review?**  For information about this choice, including consent withdrawal, please see our Privacy Policy.

Reviewer #1: No

Reviewer #2: **Yes:**  Fabrizio D'Ascenzo

**********

# Editor report: Acceptance letter

**DOI:** 10.1371/journal.pone.0344341.r006

**Authors:** Leander Klein

PONE-D-25-57504R2

PLOS One

Dear Dr. Inaba,

I'm pleased to inform you that your manuscript has been deemed suitable for publication in PLOS One. Congratulations! Your manuscript is now being handed over to our production team.

At this stage, our production department will prepare your paper for publication. This includes ensuring the following:

* All references, tables, and figures are properly cited

* All relevant supporting information is included in the manuscript submission,

* There are no issues that prevent the paper from being properly typeset

You will receive further instructions from the production team, including instructions on how to review your proof when it is ready. Please keep in mind that we are working through a large volume of accepted articles, so please give us a few days to review your paper and let you know the next and final steps.

Lastly, if your institution or institutions have a press office, please let them know about your upcoming paper now to help maximize its impact. If they'll be preparing press materials, please inform our press team within the next 48 hours. Your manuscript will remain under strict press embargo until 2 pm Eastern Time on the date of publication. For more information, please contact onepress@plos.org.

You will receive an invoice from PLOS for your publication fee after your manuscript has reached the completed accept phase. If you receive an email requesting payment before acceptance or for any other service, this may be a phishing scheme. Learn how to identify phishing emails and protect your accounts at https://explore.plos.org/phishing.

If we can help with anything else, please email us at customercare@plos.org.

Thank you for submitting your work to PLOS ONE and supporting open access.

Kind regards,

PLOS ONE Editorial Office Staff

on behalf of

Professor Leander Luiz Klein

Academic Editor

PLOS One
